# Select gp120 V2 domain specific antibodies derived from HIV and SIV infection and vaccination inhibit gp120 binding to α_4_β_7_

**DOI:** 10.1371/journal.ppat.1007278

**Published:** 2018-08-28

**Authors:** Sakaorat Lertjuthaporn, Claudia Cicala, Donald Van Ryk, Matthew Liu, Jason Yolitz, Danlan Wei, Fatima Nawaz, Allison Doyle, Brooke Horowitch, Chung Park, Shan Lu, Yang Lou, Shixia Wang, Ruimin Pan, Xunqing Jiang, Francois Villinger, Siddappa N. Byrareddy, Philip J. Santangelo, Lynn Morris, Constantinos Kurt Wibmer, Kristin Biris, Rosemarie D. Mason, Jason Gorman, Joseph Hiatt, Elena Martinelli, Mario Roederer, Dai Fujikawa, Giacomo Gorini, Genoveffa Franchini, Anush Arakelyan, Aftab A. Ansari, Kovit Pattanapanyasat, Xiang-Peng Kong, Anthony S. Fauci, James Arthos

**Affiliations:** 1 Department of Immunology, Faculty of Medicine Siriraj Hospital, Mahidol University, Bangkok, Thailand; 2 Department of Research and Development, Faculty of Medicine Siriraj Hospital, Mahidol University, Bangkok, Thailand; 3 Laboratory of Immunoregulation, National Institute of Allergy and Infectious Diseases, National Institutes of Health, Bethesda, MD, United States of America; 4 Department of Medicine, University of Massachusetts Medical School, Worcester, MA, United States of America; 5 Department of Biochemistry and Molecular Pharmacology, NYU School of Medicine, New York, NY, United States of America; 6 New Iberia Research Center and Department of Biology, University of Louisiana at Lafayette, Lafayette, LA, United States of America; 7 Department of Pharmacology and Experimental Neuroscience, University of Nebraska Medical Center, Omaha, NE, United States of America; 8 Wallace H. Coulter Department of Biomedical Engineering, Georgia Institute of Technology and Emory University, Atlanta, Georgia, United States of America; 9 Center for HIV and STIs, National Institute for Communicable Diseases of the National Health Laboratory Service (NHLS), Johannesburg, South Africa; 10 Faculty of Health Sciences, University of the Witwatersrand, Johannesburg, South Africa; 11 Centre for the AIDS Programme of Research in South Africa (CAPRISA), University of KwaZulu-Natal, Congella, South Africa; 12 Vaccine Research Center, National Institute of Allergy and Infectious Diseases, National Institutes of Health, Bethesda, MD, United States of America; 13 Microbiology and Immunology, University of California, San Francisco, CA, United States of America; 14 Center for Biomedical Research, Population Council, New York, NY, United States of America; 15 Animal Models and Vaccine Section, National Cancer Institute, National Institutes of Health, Bethesda, MD, United States of America; 16 Section on Intercellular Interactions, Eunice Kennedy-Shriver National Institute of Child Health and Human Development, National Institutes of Health, Bethesda, MD, United States of America; 17 Department of Pathology and Laboratory Medicine, Emory University School of Medicine, Atlanta, GA, United States of America; University of Zurich, SWITZERLAND

## Abstract

The GI tract is preferentially targeted during acute/early HIV-1 infection. Consequent damage to the gut plays a central role in HIV pathogenesis. The basis for preferential targeting of gut tissues is not well defined. Recombinant proteins and synthetic peptides derived from HIV and SIV gp120 bind directly to integrin α_4_β_7_, a gut-homing receptor. Using both cell-surface expressed α_4_β_7_ and a soluble α_4_β_7_ heterodimer we demonstrate that its specific affinity for gp120 is similar to its affinity for MAdCAM (its natural ligand). The gp120 V2 domain preferentially engages extended forms of α_4_β_7_ in a cation -sensitive manner and is inhibited by soluble MAdCAM. Thus, V2 mimics MAdCAM in the way that it binds to α_4_β_7_, providing HIV a potential mechanism to discriminate between functionally distinct subsets of lymphocytes, including those with gut-homing potential. Furthermore, α_4_β_7_ antagonists developed for the treatment of inflammatory bowel diseases, block V2 binding to α_4_β_7_. A 15-amino acid V2 -derived peptide is sufficient to mediate binding to α_4_β_7_. It includes the canonical LDV/I α_4_β_7_ binding site, a cryptic epitope that lies 7–9 amino acids amino terminal to the LDV/I, and residues K169 and I181. These two residues were identified in a sieve analysis of the RV144 vaccine trial as sites of vaccine -mediated immune pressure. HIV and SIV V2 mAbs elicited by both vaccination and infection that recognize this peptide block V2-α_4_β_7_ interactions. These mAbs recognize conformations absent from the β- barrel presented in a stabilized HIV SOSIP gp120/41 trimer. The mimicry of MAdCAM-α_4_β_7_ interactions by V2 may influence early events in HIV infection, particularly the rapid seeding of gut tissues, and supports the view that HIV replication in gut tissue is a central feature of HIV pathogenesis.

## Author summary

HIV is gut-tropic. Disruption of GALT plays an important role in HIV -mediated immune dysfunction. α_4_β_7_ is a receptor that facilitates homing of lymphocytes to GALT. α_4_β_7_^high^ CD4^+^ T cells are early targets of HIV infection. The HIV envelope protein gp120 binds to α_4_β_7_; however, a link between this interaction and the preferential infection of α_4_β_7_^high^ CD4^+^ T cells has not been established. Here we report an apparent gp120 mimicry of MAdCAM, a natural ligand of α_4_β_7_. That the HIV envelope protein mimics an adhesion receptor expressed primarily in gut tissues supports the concept of a central role for GALT in HIV pathogenesis. Antibodies derived from vaccination and infection were evaluated for their capacity to inhibit gp120-α_4_β_7_ interactions. Antibodies that inhibit binding target a region of the V2 domain that has been linked with reduced risk of acquisition in the RV144 vaccine trial. These antibodies recognize epitopes that do not appear on the closed gp120/41 trimer. Instead, they recognize structures that form when V2 is allowed to fold in a less constrained way. We show that such epitopes can appear on virions. Thus, an alternative structure of the V2 domain may facilitate the trafficking of HIV to GALT.

## Introduction

Gut associated lymphoid tissue (GALT) is a primary target for HIV and SIV, particularly in the early weeks of infection [1, 2]. Within days after transmission, high levels of proviral DNA can be isolated from GALT [2–4]. Subsequently, gut CD4^+^ T cells are severely depleted, and the structural integrity of GALT is to a large extent irreversibly damaged in a way that is thought to contribute to chronic immune activation [5–7]. Administration of anti-retroviral therapy (ART), even shortly after infection, fails to fully reverse this damage [8]. These early events in infection are believed to contribute in a significant way to the immune dysfunction that characterizes HIV disease [5]. Thus, the early seeding of gut tissues plays a central role in HIV pathogenesis.

We and others have demonstrated that both HIV and SIV recombinant envelope proteins directly bind integrin α_4_β_7_ (α_4_β_7_), a gut homing receptor [9–12], while notably, some studies have failed to detect this interaction [13–15]. α_4_β_7_ is not required for viral entry [15–17]. However, our findings raise the possibility that there exists a link between the gut-tropic aspect of HIV infection and this physical interaction. It is possible that α_4_β_7_ functions simply as an attachment factor [18]. However, gp120 binding to α_4_β_7_, like mucosal addressin cellular adhesion molecule (MAdCAM) transduces signals to primary CD4^+^ T cells, suggesting that such signals may be relevant to infection in vivo [9, 17, 19]. In this regard we have recently reported that MAdCAM delivers a signal to CD4^+^ T cells that promotes cellular activation and viral replication [19].

α_4_β_7_ is expressed on the cell-surface membrane of a number of cellular subsets including most naive CD4^+^ T cells and a subset of memory CCR5^+^/CD4^+^ T cells [20]. Similar to each of the 24 human integrins, α_4_β_7_ is a heterodimer. It is comprised of a 180 kDa α_4_ chain [21] and a 130 kDa β_7_ chain [22]. α_4_β_7_ is structurally dynamic and can adopt at least three conformational states, two of which are extended, and competent to mediate lymphocyte adhesion [23]. Transition between conformations is tightly controlled intracellularly, which provides a regulatory mechanism for α_4_β_7_ activity [24]. The normal function of α_4_β_7_ involves binding to two adhesion receptors, MAdCAM and vascular addressin cellular adhesion molecule (VCAM), along with the alternatively-spliced III connecting segment (CS) fragment of fibronectin [25]. Importantly, α_4_β_7_ is the only integrin capable of binding to MAdCAM [26]. In healthy adults MAdCAM is expressed on follicular dendritic cells in gut tissues [27, 28] and on endothelial cells lining the lumen of high endothelial venules in GALT and the gut lamina propria [29–31]. The specificity of MAdCAM for α_4_β_7_, along with its tissue specific expression are the two factors that define α_4_β_7_ as the gut homing integrin receptor.

There is growing evidence that α_4_β_7_ plays a significant role in the pathogenesis of HIV disease. It has been shown that α_4_β_7_^high^ CD4^+^ memory T cells are preferentially infected during both HIV and SIV acute infection [8, 32]. Additionally, the frequency of α_4_β_7_^high^ CD4^+^ memory T cells is correlated with risk of acquisition in both SIV and HIV [8, 33], and in HIV this association was shown to be independent of other markers of cellular activation [8]. Sexually transmitted diseases (STDs), which are associated with increased risk of acquisition of HIV, increase the frequency of α_4_β_7_^high^ CD4^+^ memory T cells in both genital mucosa and blood [34, 35]. In one recent study of HIV infected women, pre-infection levels of peripheral blood α_4_β_7_^high^ CD4^+^ memory T cells correlated with the rate of CD4^+^ T cell decline post-infection [8]. In an SIV rhesus macaque model, a substantial proportion of animals pretreated with an anti α_4_β_7_ monoclonal antibody (mAb) were protected from infection following repeated low-dose vaginal challenge [36]. The same mAb, when combined with ART, promoted durable immune-mediated control of viremia in SIV infected macaques after all forms of therapy were withdrawn [37]. Taken together, these findings demonstrate the importance of α_4_β_7_-expressing cells in HIV/SIV infection, and also in the ensuing host immune response, and underscore the need for a more complete understanding the role of α_4_β_7_ in the pathogenesis of HIV/SIV disease.

Previous studies have demonstrated that the carboxy-terminal region of the V2 domain of gp120 interacts with α_4_β_7_ [9, 10, 12]. Using site directed mutagenesis of gp120 we reported that a tripeptide motif leucine-aspartic acid-valine or isoleucine at positions 179–181 (L^179^D^180^V/I^181^) in the V2 domain plays a central role in this interaction. This tripeptide motif is similar to critical binding epitopes in the natural ligands of α_4_β_7_. MAdCAM encodes a leucine-aspartic acid-threonine (LDT), VCAM encodes an isoleucine-aspartic acid-valine (IDV), and the IIICS fragment of fibronectin encodes leucine-aspartic acid-valine (LDV). The key feature of each is a core aspartic acid flanked by an aliphatic residue. In each of these natural ligands the aspartic acid coordinates with a Mg^++^ ion that sits in the metal ion dependent adhesion site (MIDAS) of β_7_. Mg^++^ ion coordination is a strict requirement for ligand binding [23, 38]. Cardozo and colleagues identified nearby amino acids, glutamine-arginine-valine (QRV) (170–172) that also influence V2-α_4_β_7_ interactions, demonstrating that the binding site is not limited to the LDV/I tripeptide [12]. These two regions of V2 are flanked by potential N-linked glycosylation sites (PNGs), and removal of these flanking PNGs can enhance binding of recombinant gp120 to α_4_β_7_ [16]. It is unknown whether this enhancement is due to relief from steric hindrance, or allosteric changes in the α_4_β_7_ binding epitope. High-resolution cryo-electron microscopy and X-ray diffraction analyses indicate that the three V2 domains appear at the apex of the trimeric envelope spike [39–41]. In these structures, as well as in structures derived from scaffolded V1/V2 proteins and monomeric gp120 subunits, the region of V2 from 170–181 appears in the context of a β strand or β barrel [40–45]. In high-resolution structures, the LDV/I appears partially or fully buried in a way that would seem to make it inaccessible to α_4_β_7_. Thus, it is reasonable to conclude that the context in which α_4_β_7_ binds to V2 must involve either a rearrangement of these structures, or an alternative presentation of V2.

In this study, we characterized the physical interaction between the HIV envelope and α_4_β_7_ reasoning that such information could provide valuable insight regarding the role of α_4_β_7_-expressing cells in HIV pathogenesis. In this report, we demonstrate that a region near the carboxy-terminus of gp120 V2 appears to mimic, to a significant degree, the way in which MAdCAM engages α_4_β_7_. In this regard, MAdCAM utilizes dynamic and tightly regulated changes in the conformation of α_4_β_7_ to regulate α_4_β_7_-expressing lymphocyte access to both gut inductive and effector sites. Thus, this mimicry may provide HIV a mechanism to access gut tissues in a relatively efficient way, and argues that viral replication in gut tissues is central to HIV pathogenesis. One consequence of this mimicry is that drugs developed to antagonize MAdCAM-α_4_β_7_ interactions could also disrupt V2-α_4_β_7_ interactions. In addition, we find that a subset of HIV and SIV V2 antibodies derived from both infected subjects and vaccine recipients can effectively block V2 α_4_β_7_ interactions. Several of the vaccine -elicited weakly neutralizing mAbs have been linked with protection from infection. Rather than binding to the closed trimeric spike that is the primary target of broadly neutralizing antibodies these mAbs recognize an alternative conformation of the V2 region. This suggests that α_4_β_7_ also recognizes an alternative form of V2, that is conserved in both HIV and SIV.

## Results

### Binding vs. adhesion

To characterize the interaction between gp120 and α_4_β_7_ we employed two assays. We developed a novel surface-plasmon resonance (SPR) based assay that utilized dextran surfaces coated with recombinant envelope (env) proteins, V1/V2 scaffolds, or synthetic V2 cyclic peptides. The analyte that we reacted with these surfaces was a recombinant soluble α_4_β_7_ heterodimer in which the carboxy-terminal transmembrane and cytoplasmic tail domains of both chains were removed and replaced by short peptides that function as an “α_4_ chain acid-β_7_ chain base coiled-coil clasp” [46]. This acid-base clasp was joined by a disulfide bond that served to stabilize the heterodimer. In one iteration of this assay we employed short linear peptides derived from V2 as competitive inhibitors. The second assay we employed was a static adhesion assay based on the method developed by Peachman and colleagues, in which RPMI8866 cells, that express α_4_β_7_ on the cell surface, were allowed to adhere to the recombinant env proteins, V1/V2 scaffolds or synthetic V2 cyclic peptides (S1A Fig). The α_4_β_7_-expressing RPMI8866 cell line was derived from a human B cell lymphoma, and expresses α_4_β_7_, but no detectable CD4 or CCR5. Cells were grown in media containing retinoic acid, which increased both levels of expression, and clustering of α_4_β_7_ (S2B Fig). In some assays we included anti-integrin and anti-gp120 mAbs as adhesion inhibitors. The SPR assay allowed us to evaluate the kinetics of integrin-gp120 binding, while the cell-based assay measured adhesion between two multivalent surfaces.

### The specific affinity of α_4_β_7_ for gp120 V2 is similar to its affinity for MAdCAM

**Fig 1:**
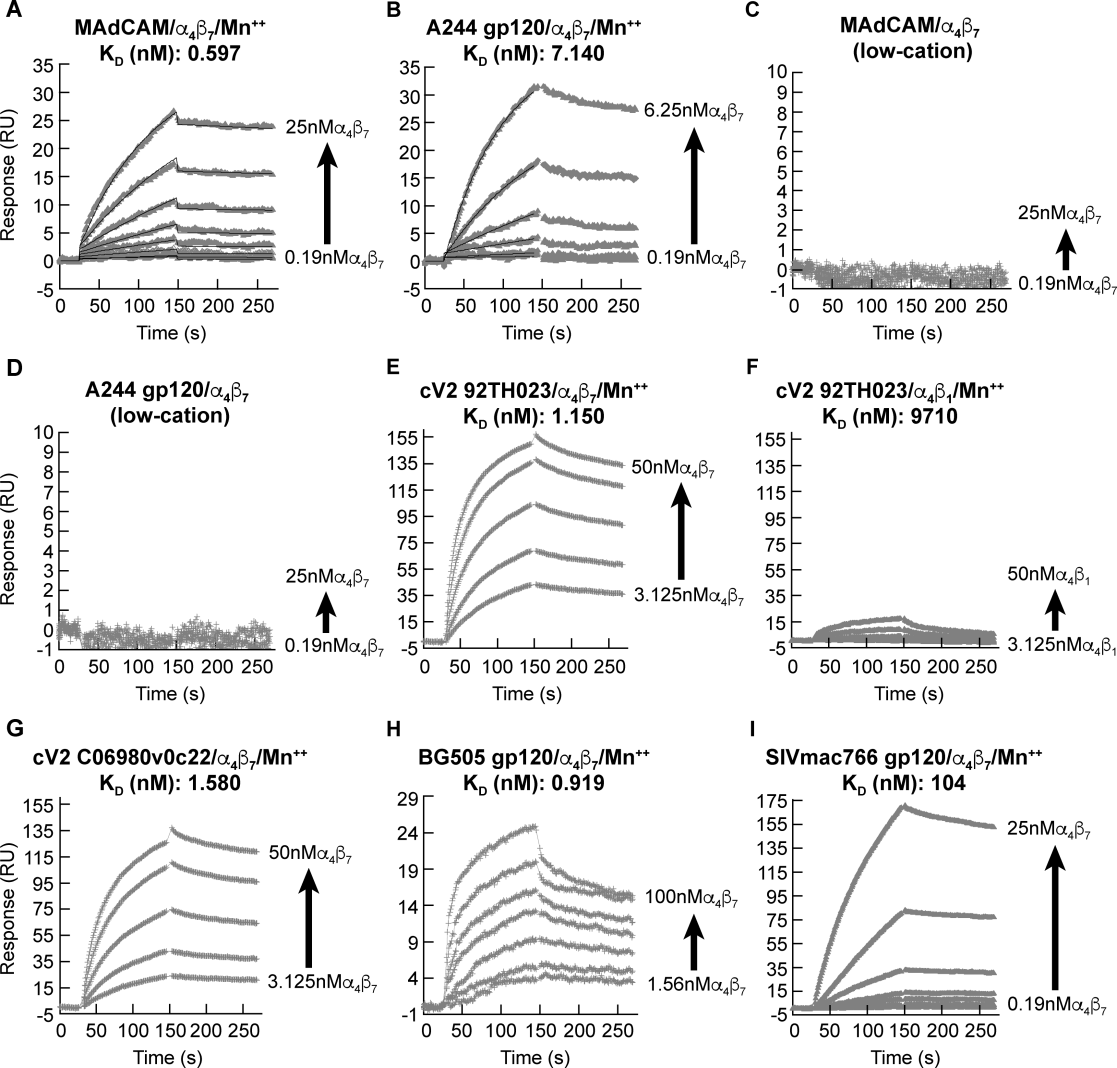
Specific affinity of α_4_β_7_ for HIV gp120, MAdCAM and SIV gp120. **A)** Sensorgram of increasing concentrations (2-fold) of soluble α_4_β_7_ passed over surface-immobilized MAdCAM in the presence of 1 mM MnCl_2_ for 120 sec, followed by a 120 sec washout/dissociation phase. Mass of bound α_4_β_7_ (y-axis) expressed as response units (RU). Affinity expressed as K_D_ (nM) is shown. **B)** Same as in panel A, with immobilized A244 gp120. **C)** Same as in panel A with immobilized MAdCAM in the absence of divalent cations. **D)** Same as in panel A with immobilized A244 gp120 in the absence of divalent cations. **E)** Same as in panel A with immobilized cV2 92TH023 peptide. **F)** Same as in panel A with immobilized 92TH023 cV2 peptide and soluble α_4_β_1_ as the analyte. **G)** Same as in panel A with immobilized cV2 C06980v0c22 peptide. **H)** Same as in panel A with immobilized BG505 gp120. **I)** Same as in panel A with immobilized SIVmac766 gp120. Additional binding parameters are listed in S1 Table. Each sensorgram is representative of three independent measurements of each analyte-ligand interaction.

Previous studies describing the interaction between gp120 and α_4_β_7_ have demonstrated, in a qualitative way, the specific interaction between these two proteins, without establishing an estimate of affinity [9–12]. We reasoned that a quantitative comparison of the binding kinetics of gp120 vs. MAdCAM to α_4_β_7_ would help determine if α_4_β_7_-gp120 interactions mimic α_4_β_7_-MAdCAM interactions. We carried out a kinetic analysis using the SPR assay noted above. Soluble α_4_β_7_ (analyte) was passed over surfaces coupled with either recombinant gp120 or a MAdCAM-Ig fusion protein (ligands). A recombinant A244 gp120 (subtype A/E produced by Global Solutions for Infectious Diseases (GSID)) was employed in these assays. We initially measured binding kinetics in the presence of a buffer containing 1mM MnCl_2_ in order to uniformly stabilize α_4_β_7_ in an extended/activated conformation (discussed below). Under these conditions MAdCAM and A244 gp120 demonstrated comparable high affinities (*K*_*D*_ (nM)) of 0.597 and 7.140, respectively (Fig 1A and 1B). These affinities are generally comparable to gp120-soluble CD4 binding kinetics (e.g 22 nM *K*_*D*_) [47]. When MnCl_2_ was removed, affinity for both MAdCAM and gp120 fell below the detection limit of this assay (Fig 1C and 1D). This requirement for Mn^++^ is consistent with our previous report that gp120, similar to MAdCAM, interacts with an extended conformation of α_4_β_7_ [9]. This observation suggests that gp120 is likely to engage α_4_β_7_ only on cells with an enhanced potential to traffic to the gut. We next replaced A244 gp120 with a synthetic cyclic 42 amino acid peptide fragment (cV2) derived from the V2 domain (AA 157–196 (HXB2 numbering)) of 92TH023 gp120 (subtype A/E), in which N and C termini were joined by a disulfide bond, and the C terminus was biotinylated to facilitate coupling to NeutrAvidin coated biosensor chips. The 92TH023 V2 sequence is nearly identical to that of A244 gp120 V2 (S1A Fig). The affinity of this peptide (cV2 92TH023) for α_4_β_7_ (*K*_*D*_ (nM) 1.150) was close to that of A244 gp120 (Fig 1E), demonstrating that a cV2 is sufficient to mediate the high-affinity interaction shown in Fig 1B. Moreover, it indicates that this high-affinity interaction does not require the glycans that decorate the V2 of GSID A244 gp120, or any bridging protein [13]. When we replaced α_4_β_7_ with α_4_β_1_ affinity for cV2 92TH023 was reduced by >8000 -fold ((*K*_*D*_ (nM) 9710) (Fig 1F), demonstrating binding specificity and the fact that V2, like MAdCAM, preferentially binds to α_4_β_7_.

**Fig 2:**
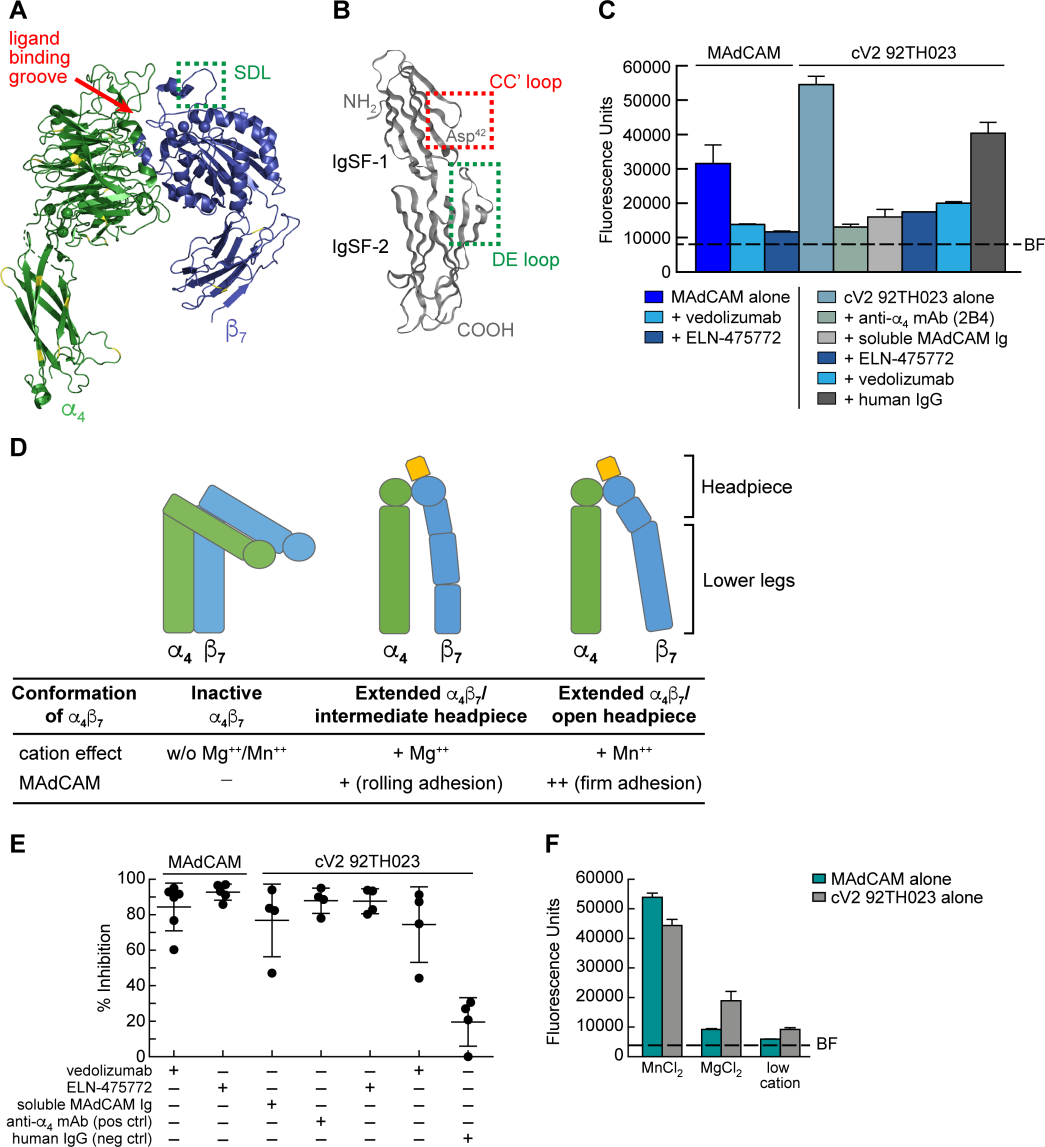
Inhibition of α_4_β_7_ -mediated cell adhesion to MAdCAM and a cyclic V2 peptide. **A)** Ribbon diagram of a human α_4_β_7_ heterodimer headpiece (α_4_: green, β_7_: blue) (PDB ID: 3V4P). Yellow indicates sequence divergence in rhesus macaque. Ligand binding groove is highlighted by a red arrow, and the SDL is highlighted by a green box. **B)** Ribbon diagram of the two N-terminal IgSF domains of human MAdCAM (PDB ID: 1GSM). The MAdCAM CC′ loop of IgSF domain 1 is highlighted in the red box and the DE loop of IgSF domain 2 is highlighted in a green box. **C)** A representative result of RPMI8866 cells adhering to immobilized MAdCAM or a cV2 92TH023 peptide in the absence or presence of vedolizumab, an LDV mimetic ELN-475772, the anti-α_4_ specific mAb 2B4, soluble MAdCAM-Ig, or control human IgG, as indicated. Adhesion was determined at OD_590nm_ and listed as fluorescence units (y-axis). Background fluorescence (BF) of RPMI8866 cell adhesion to blank wells is denoted by a dashed line. Adhesion to immobilized MAdCAM serves as a specificity control for α_4_β_7_ adhesion and human IgG is employed as a reagent control. Conditions are run in triplicate and error bars indicate standard error of the mean (SEM). **D)** Schematic of three conformations of the cell surface expression of the α_4_β_7_ heterodimer. The influence of divalent cations (Mg^++^ and Mn^++^) on the type of adhesion is listed below. **E)** Adhesion of RPMI8866 cells to either immobilized MAdCAM or cV2 92TH023 as in panel C. Average results from four or more independent experiments are shown. Y-axis indicates % inhibition relative to either MAdCAM or cV2 92TH023 in the absence of any inhibitor. Inclusion of inhibitors is indicated by a + sign below. Error bars indicate standard deviation (SD). **F)** Adhesion of RPMI8866 cells to immobilized MAdCAM or a cV2 92TH023 peptide in the buffers containing a low concentration of divalent cations, or high concentrations of MnCl_2_ or MgCl_2_. Adhesion was determined at OD_590nm_ and listed as fluorescence units (y-axis). Conditions are run in triplicate and error bars indicate standard error of the mean (SEM). Background fluorescence (BF) of RPMI8866 cells to blank wells is denoted by a dashed line. Two additional replicate experiments are shown in S3 Fig.

We extended this analysis by measuring binding kinetics for a cV2 peptide derived from C06980v0c22 (subtype C) and a recombinant gp120 BG505 protein (subtype A) (Fig 1G and 1H). α_4_β_7_ bound to each with similar high affinities, consistent with the conserved nature of this interaction across HIV clades as we had originally reported [9]. The V2 domains of HIV and SIV diverge both in primary sequence, as well as in length and the typical number of disulfide bridges (1 vs 2), suggesting significant structural differences between them. Using a recombinant gp120 derived from SIVmac766 we obtained an α_4_β_7_ affinity (*K*_*D*_ (nM)) of 104 (Fig 1I). This is ~10-fold lower than that observed for HIV A244 gp120. It is possible that this reduction in affinity reflects differences in the primary amino acid sequences of human vs. rhesus macaque α_4_β_7_ (Figs 2A and S1A), such that SIV gp120s might exhibit a higher affinity for a rhesus macaque versus a human derived α_4_β_7_ protein. Detailed α_4_β_7_ binding parameters for each of the analytes appears in S1 Table.

### α_4_β_7_ antagonists developed to block MAdCAM binding also block gp120 V2 adhesion

Similarities between MAdCAM and HIV gp120 with respect to affinity, cation-dependence, and preference for binding α_4_β_7_ over α_4_β_1_ suggests that gp120 V2 mimics MAdCAM in the manner in which it engages α_4_β_7_. A number of α_4_β_7_ antagonists, developed to treat inflammatory bowel disease (IBD) act by occupying MAdCAM binding sites on α_4_β_7_. To the extent that gp120 uses these same sites, such antagonist should also interfere with gp120 adhesion. These antagonists include a class of small molecule mimetics that resemble the Leu-Asp binding motif present in the CC′ β strands of MAdCAM IgSF domain 1 [48]. They compete directly with MAdCAM by binding to key residues in the ligand binding groove formed by the interface between α_4_ and β_7_ (Fig 2A). Vedolizumab is a mAb antagonist of α_4_β_7_ with a different and unique mechanism of action (MOA). It binds exclusively to the specificity-determining loop (SDL) in β_7_ (Fig 2A) [23]. The SDL mediates secondary interactions with a negatively charged DE loop in MAdCAM IgSF domain 2 (Fig 2B) [23, 49–52]. We evaluated the capacity of a small molecule LDV mimetic, ELN-475772, and vedolizumab to interfere with gp120-α_4_β_7_ interactions using the RPMI8866-based adhesion assay described above. As expected, both ELN-475772 and vedolizumab blocked adhesion of α_4_β_7_ to immobilized MAdCAM (Fig 2C and 2E). We then tested their ability to block α_4_β_7_-mediated adhesion to the cV2 92TH023. The anti-α_4_ mAb 2B4, which inhibits most α_4_-ligand interactions, along with human IgG were employed as specificity controls. Both ELN-475772 and vedolizumab inhibited α_4_β_7_ adhesion to V2 by >90% (Fig 2C and 2E). Because the inhibitory MOAs of ELN-475772 and vedolizumab involve direct interactions with two discreet MAdCAM binding sites on α_4_β_7_, these results suggest that soluble MAdCAM would compete with gp120 V2 in binding to α_4_β_7_. Of note, soluble MAdCAM-Ig blocked V2-α_4_β_7_ adhesion (Fig 2C and 2E). We conclude that, at least in a general way, gp120 V2 effectively mimics MAdCAM in the manner in which it engages α_4_β_7_.

α_4_β_7_ activity is regulated by its conformation. Intracellular signaling events modulate the ectodomain to reversibly transition between bent and extended conformations (Fig 2D). In addition, the headpiece, which mediates ligand interactions, can assume closed, intermediate, and open conformations. Transitions between these conformations allows α_4_β_7_ to mediate both rolling and firm adhesion [23]. Rolling adhesion is associated with lower affinity binding to MAdCAM, while firm adhesion is associated with a higher affinity interaction. Manipulation of Ca^++^, Mg^++^, and Mn^++^ concentrations provides a way to manipulate the affinity of α_4_β_7_-MAdCAM interactions in vitro [38]. Strength of adhesion is highest in Mn^++^, followed by Mg^++^ > Mg^++^/Ca^++^, > Ca^++^. We asked whether the pattern of V2 adhesion to α_4_β_7_ was similar to that mediated by MAdCAM in buffers containing Mn^++^ vs Mg^++^, vs low cations (Ca^++^). Adhesion of both MAdCAM and cV2 92TH023 was strongest in the presence of MnCl_2_ and reduced in the presence of MgCl_2_ by ~5-fold and 2.5-fold respectively (Figs 2F and S3). In low-cation conditions MAdCAM adhesion appeared close to background levels; however, we were still able to detect residual cV2 92TH023 adhesion. Overall, V2 adhesion to α_4_β_7_ responded to divalent cations in a similar manner as did MAdCAM. This is particularly noteworthy insofar as the dynamic changes in affinity required to mediate both rolling and firm adhesion reflect the unique and highly specialized nature of α_4_β_7_-MAdCAM interactions [23]. The way in which both HIV and SIV gp120 mimic this highly specialized interaction argues that it provides them a selective advantage.

### Weakly neutralizing HIV V2 mAbs elicited by vaccination and infection inhibit gp120-α_4_β_7_ adhesion

**Fig 3:**
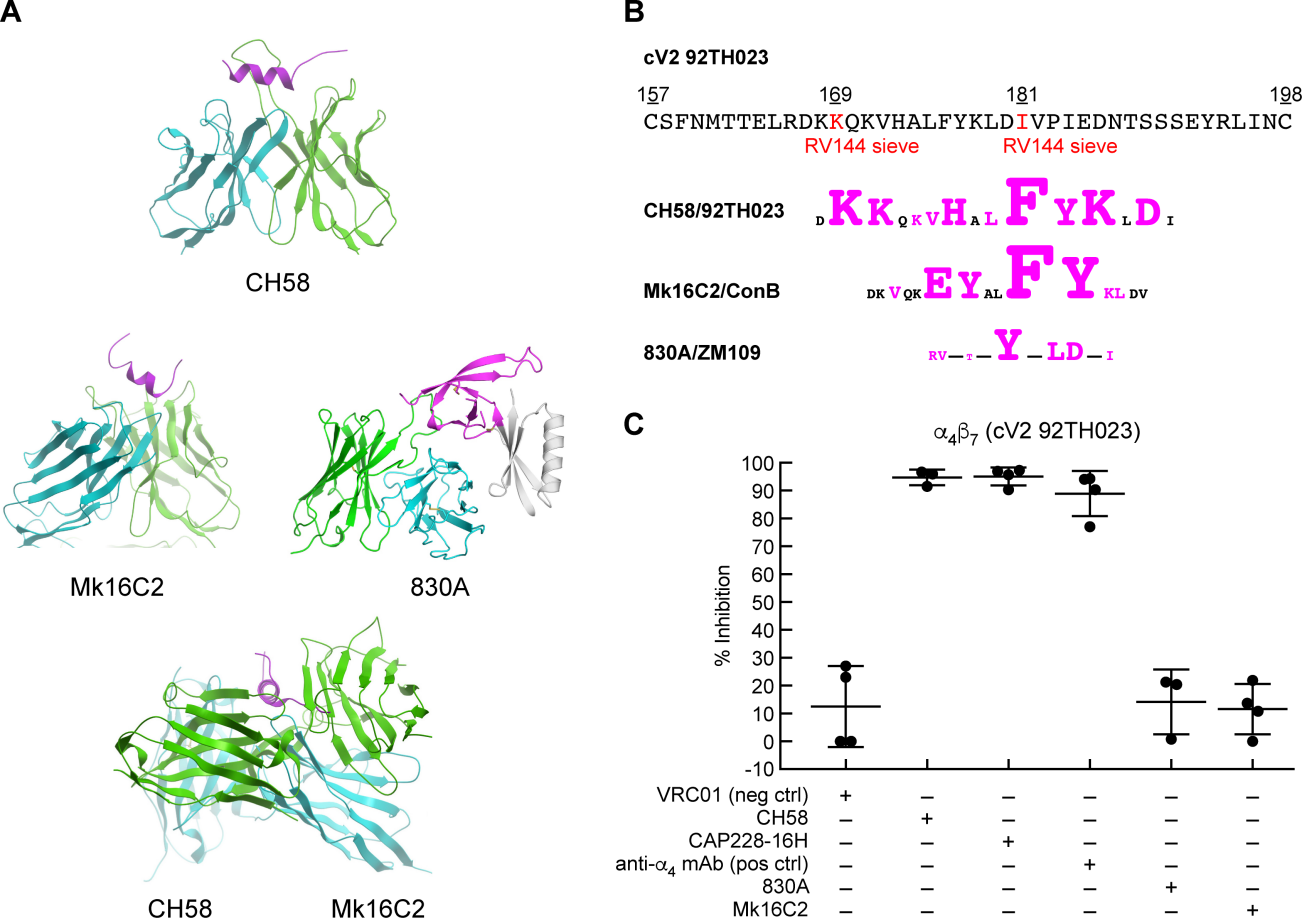
The structures of V2 mAbs in complex with peptides, and V2 mAb inhibition of α_4_β_7_ adhesion. **A)** Crystal structure of three V2 domain mAbs, CH58 (PDB ID: 4HPO), Mk16C2 (PDB ID: 6CEZ), and 830A (PDB ID: 4YWG) in complex with V2 peptides, and a superimposition of CH58 and Mk16C2 binding to opposite sides of the same helical region of a V2 peptide. Only the Fv regions are shown with the heavy chain, light chain, and the V2 epitope colored cyan, green, and magenta, respectively. **B)** Amino acid sequence of the V2 domain of 92TH023. Sieve residues identified in the RV144 vaccine study are highlighted in red, along with schematics of the epitopes of CH58, Mk16C2 and 830A aligned below (with the corresponding HIV isolate listed). Residues in contact with each mAb are highlighted in magenta and the size of each amino acid is proportional to the contact surface area. **C)** Adhesion of RPMI8866 cells to cV2 92TH023 in the absence or presence of V2 mAbs: CH58, CAP228-16H, 830A, and Mk16C2. The mAb VRC01 is included as a nonspecific mAb control, and the anti-α_4_ mAb 2B4 is employed as a positive control. Adhesion was determined at OD_590nm_ and listed as % inhibition in three or more independent experiments relative to cV2 92TH023 in the absence of any inhibitor (y-axis). Error bars indicate SD.

The conformation of the V2 domain of gp120 is dynamic, and consequently it was deleted from the recombinant proteins used to generate the initial high-resolution gp120-mAb cocrystals [53, 54]. Instead, V2 structures were obtained by grafting V1/V2 fragments onto scaffolds derived from unrelated proteins. These scaffolds stabilized V1/V2 in a way that, in complex with conformation-dependent V2 mAbs, allowed for the derivation of high resolution structures. The first V2 structure was obtained by presenting V1/V2 on a scaffold termed 1FD6 in complex with the broadly neutralizing, glycan dependent monoclonal antibody PG9 [43]. In this context, V2 adopted a Greek key β sheet structure (Fig 2A). Another study in which the same 1FD6-V1/V2 protein was complexed with mAb 830A provided additional detail and revealed V2 in a related β- barrel conformation [42], which is consistent with what has been observed in pre-fusion env trimers [40, 41]. However, when a linear V2 peptide is left unconstrained it can adopt α helical structure [55]. This is the case for a crystal structure of mAb CH58 in complex with a linear V2 peptide derived from HIV isolate 92TH023 gp120 [56]. CH58 does not exhibit broad potent neutralizing activity and we refer to here as weakly- neutralizing. It recognizes a helix structure (Fig 3A) and is noteworthy insofar as it was generated from an uninfected immunized individual who participated in the RV144 vaccine trial. It recognizes an epitope that maps within a short region of V2 (AA 168–181), that includes two residues, K^169^ and I^181^, identified by sieve analysis as sites of vaccine elicited immune pressure in the RV144 trial (Fig 3B). Additional V2-specific mAbs that recognize helical structures in this same region have subsequently been described. We report here the cocrystal structure of a V2 peptide with one such mAb, Mk16C2, that was generated from a gp120 immunized rabbit (Fig 3A and S2 Table). It binds to the same helical structure as CH58 but approaches V2 from the opposite side. Of note, helix -preferring V2 antibodies are not limited to vaccine elicited immune responses. mAb CAP228-16H, which was derived from an HIV-infected subject, recognizes a V2 helix structure that is strikingly similar to that recognized by CH58 [57]. Thus, mAbs reacting with the region of V2 from AA 153–194 recognize at least two distinct types of epitopes: those like PG9 and 830A that recognize a constrained β- sheet, and those like CH58 that recognize a less constrained helical conformation.

In an ELISA assay mAbs 830A, CH58, CAP228-16H and Mk16C2 bind to cV2 92TH023 (S1B Fig), indicating that this cyclic peptide is sufficiently long and flexible to present the epitopes recognized by each of these mAbs. Although the core epitopes of CH58 and 830A differ, the C-terminal end of the CH58 epitope overlaps the 830A epitope (Fig 3B), which is consistent with our observation that CH58 can compete with 830A (S1C Fig). We evaluated the ability of each of these mAbs to inhibit α_4_β_7_ interactions with V2 using the adhesion assay described above. mAbs 2B4 and VRC01 were employed as specificity controls. CH58 and CAP228-16H inhibited α_4_β_7_ adhesion to V2 by >90% (Fig 3C). Mk16C2 inhibited adhesion less effectively. Of note, 830A failed to inhibit adhesion in a significant way. Thus, while a V2 mAb (CH58) that recognizes a helical structure interfered with α_4_β_7_ -mediated adhesion, mAb 830A, that shows preference for the β strand, failed to show detectable interference.

### α_4_β_7_ adhesion is mediated by an unconstrained form of gp120 V2

**Fig 4:**
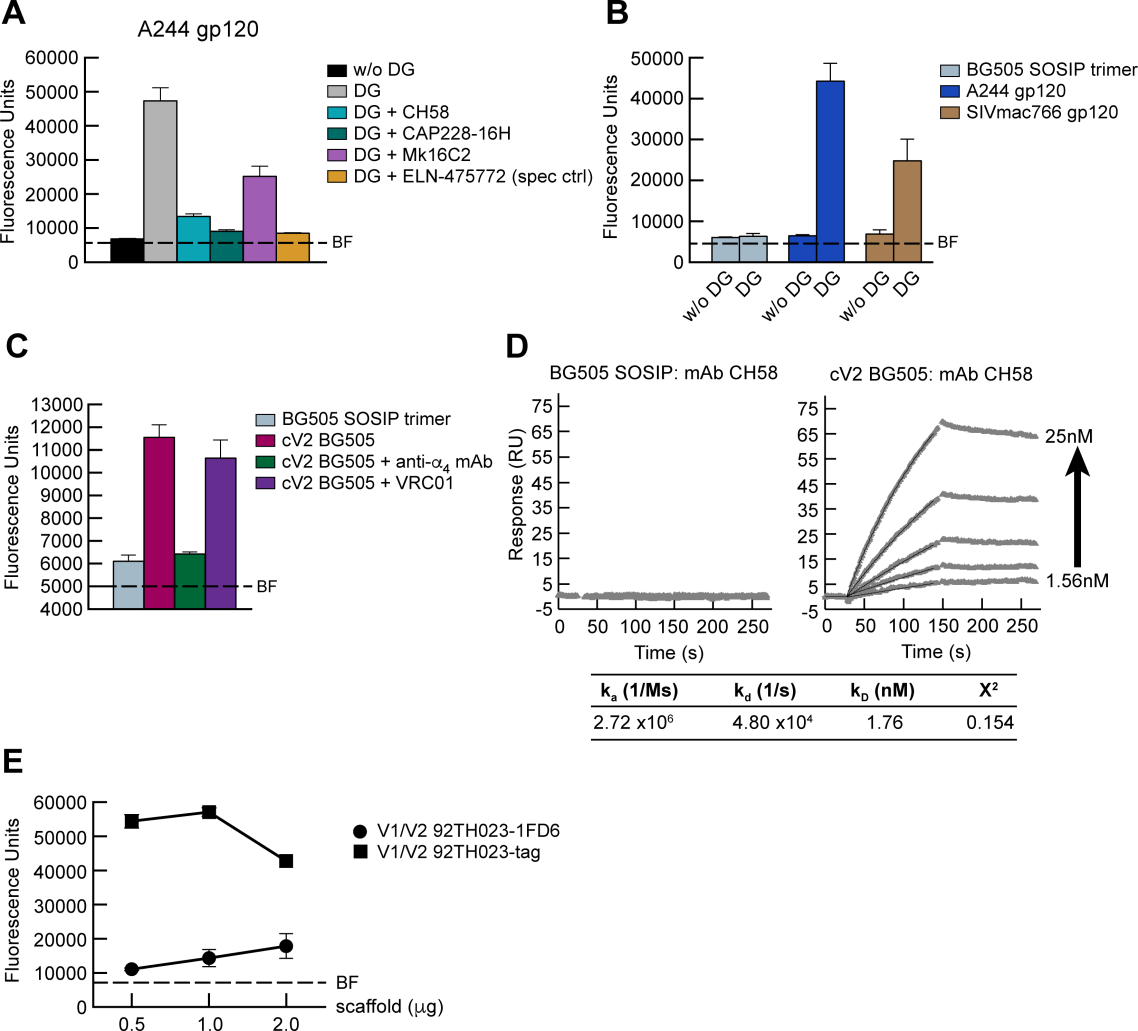
Adhesion of α_4_β_7_ to recombinant gp120 and the V1/V2 scaffolds. **A)** Adhesion of RPMI8866 cells to immobilized A244 gp120 in the absence or presence of HIV V2-specific mAbs: CH58, CAP228-16H, and Mk16C2. The LDV mimetic ELN-475772 provides a specificity control for adhesion to α_4_β_7_. Adhesion was determined by OD_590nm_ and listed as fluorescence units (y-axis). Conditions are run in triplicate and error bars indicate SEM. Background fluorescence (BF) of RPMI8866 cells is denoted by a dashed line. Deglycosylation (DG) of A244 gp120 as indicated. Results from three or more independent experiments are shown in S4 Fig. **B)** Comparison of α_4_β_7_ adhesion to glycosylated vs. DG forms of BG505 SOSIP trimer, A244 gp120, and SIVmac766 gp120 as described in panel A. Conditions are run in triplicate and error bars indicate SEM. BF is denoted by a dashed line. DG as indicated. Results from three independent experiments are shown in S5 Fig. **C)** Comparison of adhesion of RPMI8866 cells to BG505 SOSIP vs. cV2 BG505 peptide as described for panel A. The anti-α_4_ mAb 2B4 is employed as a positive control and the gp120 mAb VRC01 is employed as a negative control. Error bars indicate SEM. This result is representative of three or more independent experiments as shown in S6 Fig. **D)** SPR analysis showing increasing concentrations of mAb CH58 (2-fold increases from 1.56 nM-25 nM) binding to BG505 SOSIP and cV2 BG505 coated surfaces. The parameters for the binding kinetics for cV2 BG505 are listed. **E)** Comparison of adhesion of RPMI8866 cells to V1/V2 92TH023-1FD6 vs. V1/V2 92TH023-tag scaffolds using increasing concentrations of each scaffold as indicated (x-axis). Adhesion was determined as describe for panel A. Conditions are run in triplicate and error bars indicate SEM. Results are representative of three independent experiments.

Rao and colleagues have recently reported that adhesion of recombinant gp120 to α_4_β_7_ requires the partial enzymatic removal of glycans [58]. This is consistent with our finding that removal of several potential N-glycosylation sites (PNGs) within V2 can enhance gp120 binding to α_4_β_7_ [16]. In agreement with these reports we find that GSID A244 gp120 required limited deglycosylation with PNGase, under nonreducing conditions, in order to mediate α_4_β_7_ adhesion (Fig 4A). This adhesion, similar to the adhesion of α_4_β_7_ to cV2 92TH023, was efficiently inhibited by ELN-475772, CH58, and CAP228-16H (>90%) (Figs 4A and S4). Again, Mk16C2 was less effective (~47% reduction). We also evaluated α_4_β_7_ adhesion to a BG505 SOSIP gp120/41 trimer and monomeric SIVmac766 gp120. Unlike the two monomeric gp120s, the SOSIP stabilized protein failed to mediate adhesion after removal of glycans (Figs 4B and S5). Inability to adhere to α_4_β_7_ is not a consequence of the primary sequence of BG505 V2 since a BG505 cyclic V2 (cV2 BG505) was able to mediate α_4_β_7_ adhesion (Figs 4C and S6). These finding are in agreement with the observations of Rao and colleagues. Of note, in an SPR -based assay, CH58 binds to the cV2 BG505 but not to the BG505 SOSIP (Fig 4D). Given that CH58, which recognizes a helix, efficiently blocks V2 adhesion to α_4_β_7_, we hypothesized that the failure of the BG505 SOSIP trimer to engage α_4_β_7_ reflects an underlying conformational constraint on V2 mediated by the SOSIP stabilization strategy in which this constraint precludes the formation of a structure required for α_4_β_7_ reactivity.

To address whether the β- barrel conformation of V2 is incompatible with α_4_β_7_ -reactivity, we inserted the V1/V2 sequences of 92TH023 into two scaffolds. The first scaffold, termed 1FD6, has been previously shown to constrain V1/V2 in a way that increases its propensity to form a β- barrel [42]. The second scaffold, termed tag, consists of V1/V2 that is untethered at the C-terminus, allowing it to adopt an unconstrained, CH58 -reactive, helical conformation [42] [PMID: 27707920]. We found that the deglycosylated 92TH023 V1/V2 tag scaffold showed ~5x greater adhesion than did deglycosylated 92TH023 V1/V2 1FD6, which mediated only minor levels of α_4_β_7_ adhesion (Fig 4E). We conclude that α_4_β_7_ -reactivity requires a degree of V1/V2 flexibility that is not present in the recombinant BG505 SOSIP trimer. Our results suggest that this is due to constraints placed on V2 by other sequences encoded in the closed trimer. However, we cannot rule out interference by PNGase resistant glycans.

### α_4_β_7_ -coated nanoparticles capture HIV virions derived from primary CD4^+^ T cells

**Fig 5:**
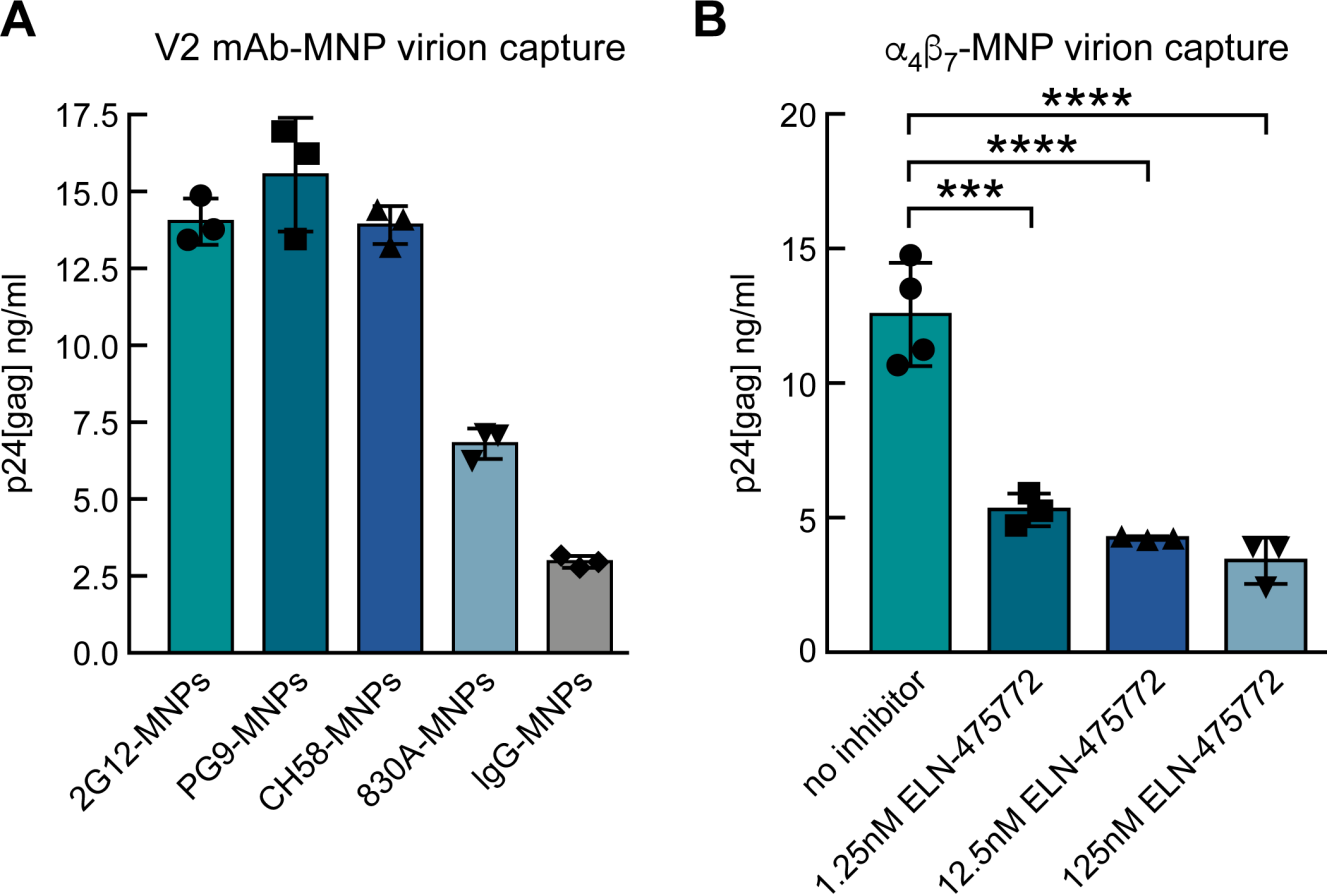
Virion capture by V2 mAbs and α_4_β_7_ coated nanoparticles. **A)** Capture of 92TH023 by MNPs coated with gp120 mAbs including 2G12 (positive control) and V2 mAbs PG9, CH58 and 830A. A non-specific Ig was employed as a negative control. Virion capture was determined by detection of MNP-bound p24 (Y-axis). Results from three independent experiments are shown. **B)** 92TH023 virion capture by α_4_β_7_-MNPs in the absence or presence of increasing concentrations of ELN-475772. Results from three independent experiments are shown. Detection of MNP -captured virus as in panel A. Error bars indicate SEM. Significance measure by ANOVA followed by the Tukey multiple comparison test (***p <0.001 and ****p <0.0001).

The data presented above suggests that α_4_β_7_ recognizes a structure distinct from the closed spike on virions that is the target of many well characterized neutralizing mAbs. However, it is well established that env appearing on virions is conformationally heterogeneous [59]. We asked whether, among these various env conformations was one that is α_4_β_7_ -reactive. To address this question, we employed magnetic nanoparticles (MNPs) coated with either α_4_β_7_ or V2 mAbs CH58, PG9, and 830A to capture 92TH023 virions derived from primary CD4^+^ T cells (Fig 5). mAb 2G12 was employed as a positive control. After extensive washing, virion capture was measured by a Luminex -based p24 detection assay [60]. In three independent experiments CH58, PG9, and 2G12 each captured ~5X greater amounts of virus than non-specific IgG. Capture by mAb 830A was less efficient (~3X over IgG) (Fig 5A). The capacity of mAb CH58, which recognizes a helical structure and inhibits V2 adhesion to α_4_β_7_ is able to capture virions suggesting that these virions present an α_4_β_7_ -reactive form. To test this directly we incubated virions with α_4_β_7_- MNPs in the absence or presence of increasing amounts of the α_4_β_7_ inhibitor ELN-475772. In three independent experiments α_4_β_7_- MNPs captured virus, and this capture was inhibited by ELN-475772 in a dose -dependent manner (Fig 5B). Thus, 92TH023 virions derived from primary CD4^+^ T cells present gp120 in an α_4_β_7_ -reactive form.

### A 15- amino acid linear V2 -derived peptide is sufficient to mediate both HIV and SIV gp120 binding to α_4_β_7_

**Fig 6:**
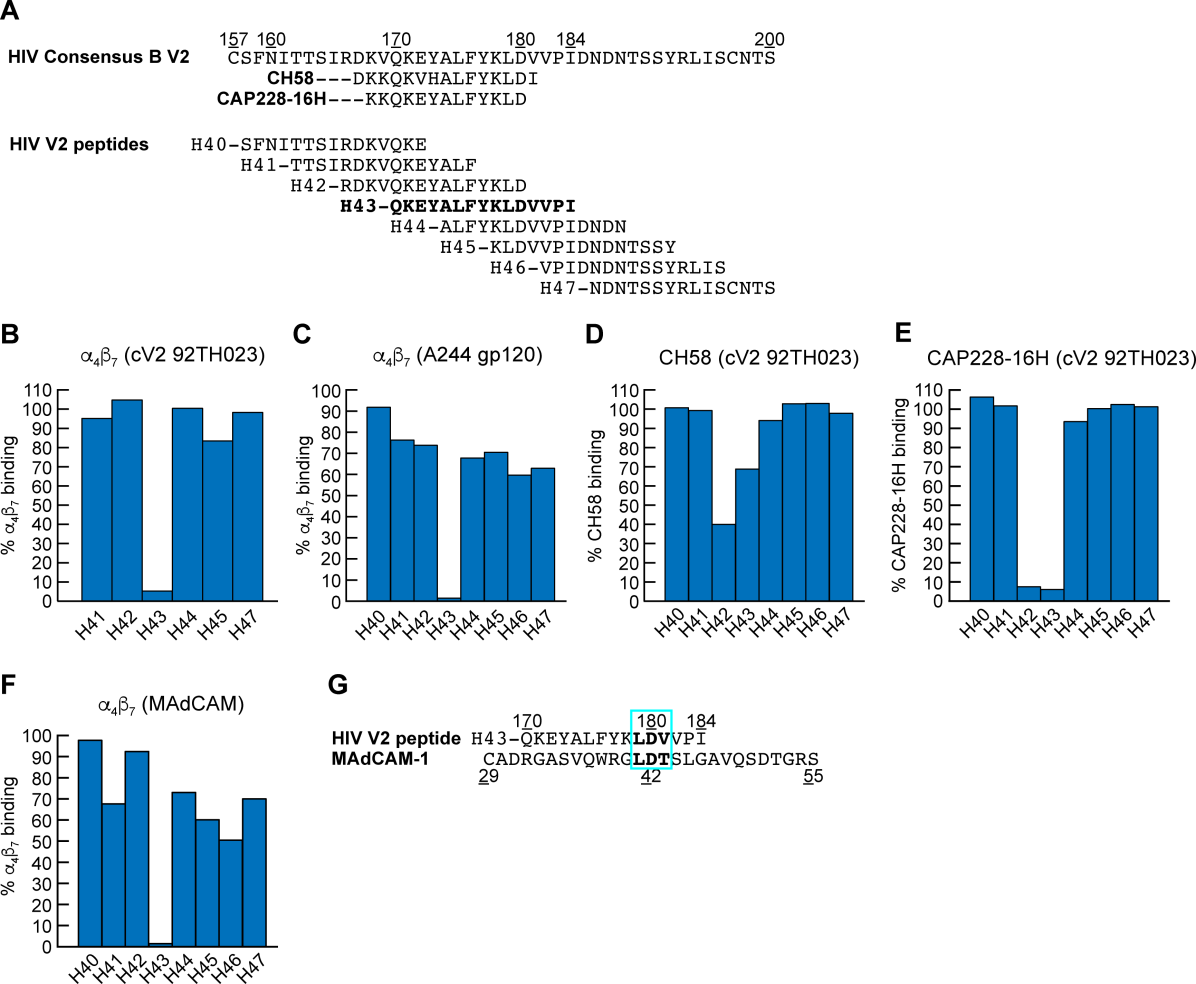
Inhibition of α_4_β_7_ binding of HIV V2 peptides to HIV gp120, cyclic V2 and MAdCAM. **A)** Alignment of the V2 domains of HIV consensus B, and the eight HIV consensus B 15mer peptides (H40-H47) used in this study, and the regions of V2 recognized by mAbs CH58 and CAP228-16H. **B-C)** HIV 15mer peptide inhibition of α_4_β_7_ binding to cV2 92TH023 and A244 gp120 as measured by SPR. Percent of α_4_β_7_ binding relative to binding in the absence of peptide (defined as 100%) is shown (y-axis). **D-E)** HIV 15mer peptide inhibition of V2 mAbs, CH58 and CAP228-16H binding to cV2 92TH023. Percent binding relative to binding in the absence of peptide is shown (y-axis). **F)** HIV 15mer peptide inhibition of α_4_β_7_ binding to MAdCAM. Percent α_4_β_7_ binding relative to binding in the absence of peptide is shown (y-axis). Results are representative of three or more independent experiments. **G)** Alignment of α_4_β_7_ binding site in MAdCAM IgSF domain 1, and HIV peptide H43. Conserved LDV/LDT binding motifs are boxed in blue.

Liao and colleagues demonstrated that a 15 AA peptide corresponding to residues 168–181 of V2 adopts a helical structure when complexed with mAb CH58 (Fig 3A) [56]. Using the SPR assay described above we asked whether a similar 15 AA peptide was sufficient to bind to α_4_β_7_. Soluble α_4_β_7_ was passed over a cV2 92TH023 coated surface in the absence or presence of 8 overlapping linear 15 amino acid peptides. These peptides corresponded to sequences in an HIV-1 subtype B consensus V2 domain (Fig 6A). Peptide H43 (Q^170^KEYALFYKLDVVPI^184^), which closely aligns with the peptide employed by Liao and colleagues, inhibited α_4_β_7_ -binding by >90% (Fig 6B). This peptide includes both the canonical L^179^D^180^ α_4_β_7_ binding site and a Q^170^K^171^E^172^ that aligns with the QRV cryptic α_4_β_7_ epitope identified by Cardozo and colleagues [12]. We repeated this analysis but substituted cV2 92TH023 with A244 gp120 and obtained a similar result (Fig 6C). We then competed mAbs CH58 and CAP228-16H with these same peptides (Fig 6D and 6E). Peptides H42 (R^166^DKVQKEYALFYKLD^180^) and H43 each partially inhibited mAb CH58 binding and strongly inhibited (>90%) CAP228-16H binding. Taken together these results suggest that inhibition by peptide H43 involves direct binding to α_4_β_7_. To rule out allosteric inhibition (i.e. peptide H43 binding directly to, and altering the conformation of V2), we carried out a similar peptide inhibition assay with immobilized MAdCAM and determined that H43 inhibited α_4_β_7_ binding to MAdCAM by >90% (Fig 6F). This result is best explained by direct competition between the Leu^179^-Asp^180^ in peptide H43 and the critical Leu^41^-Asp^42^ encoded within MAdCAM IgSF domain 1 (Fig 6G). However, we believe it is very likely that other residues in H43 also engage α_4_β_7_. We conclude that an epitope contained within a linear peptide corresponding to residues 170–181 of V2 binds directly to α_4_β_7_. This same region of V2 overlaps the epitopes recognized by CH58 and CAP228-16H.

The α_4_β_7_ binding epitope of V2 is conserved in SIV as exemplified by the specific affinity of

**Fig 7:**
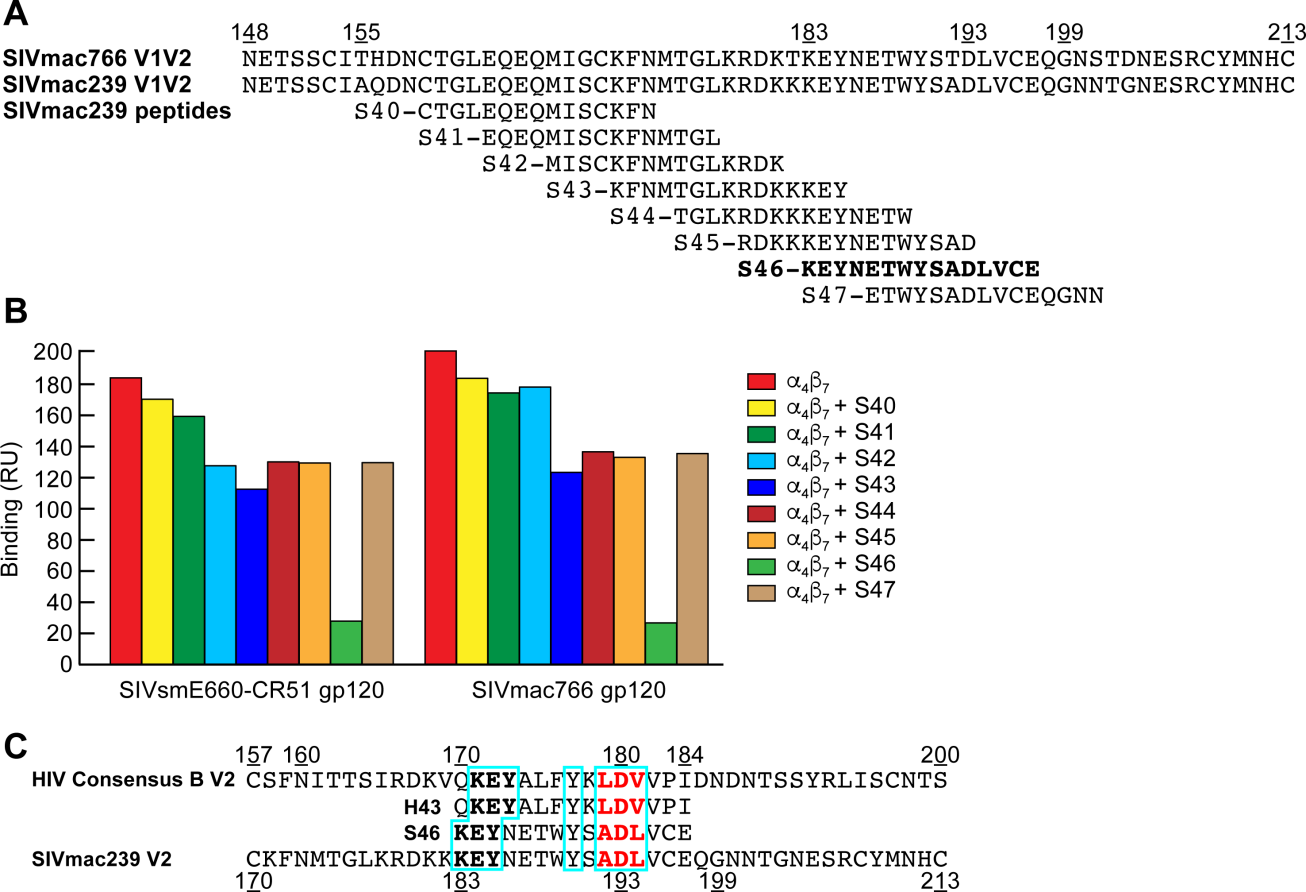
Inhibition of α_4_β_7_ binding to SIV gp120 by V2 peptides. **A)** Alignment of the V1V2 domains of SIVmac766 and SIVmac239, and eight overlapping 15 mer peptides of SIVmac239 (S40-S47). **B)** Inhibition of soluble α_4_β_7_ binding to SIVsmE660-CR51 and SIVmac766 gp120s by overlapping SIV 15mer peptide. Binding was determined by SPR analysis and binding RU at 200 seconds following the addition of α_4_β_7_ is listed (y-axis). Results are representative of three or more independent experiments **C)** Alignment of the V2 domains of HIV consensus B and SIVmac239, and peptides H43 and S46. The tri-peptide motifs (aliphatic-Asp-aliphatic residue) are highlighted in red and regions of sequence identity are boxed in blue.

SIVmac766 gp120 for human α_4_β_7_ (Fig 1I). To determine whether this region in SIV V2 is also involved in α_4_β_7_ -binding, we carried out a similar competition binding experiment as described above in Fig 6, but utilized SIVmac239 derived V2 overlapping peptides, and immobilized SIVmac766 gp120, along with SIVsmE660-CR51 gp120 (Fig 7A). Of the eight SIV peptides we employed only S46 showed strong inhibition (>90%) (Fig 7B). S46 is the SIVmac239 V2 domain peptide that corresponds to HIV peptide H43 (Fig 7C). Peptides H43 and S46 show limited sequence identity, but notably 5 residues: K^171/183^, E^172^/^184^, Y^173^/^185^, Y^177/190^, D^180/193^, appear to be conserved (Fig 7C).

**Fig 8:**
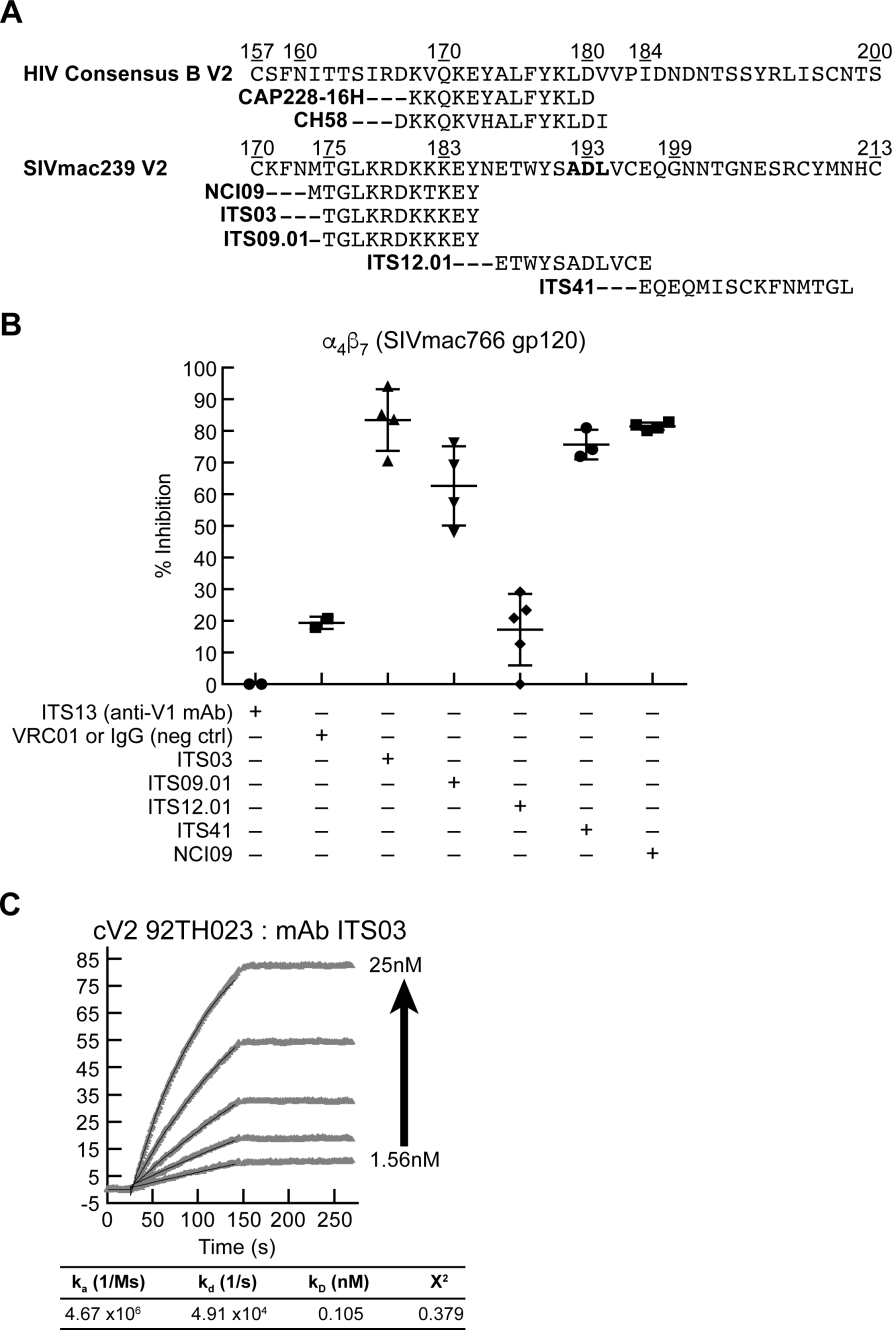
Inhibition of α_4_β_7_ adhesion by SIV V2 specific mAbs. **A)** Alignment of the amino acid sequences of the V2 domains of HIV consensus B and SIVmac239, and the regions of the V2 containing epitopes recognized by V2 specific mAbs. **B)** Adhesion of RPMI8866 cells to DG SIVmac766 gp120 in the presence of SIV V2-specific mAbs: ITS03, ITS09.01, ITS12.01, ITS41, and NCI09. ITS13, a V1 mAb and VRC01, an HIV gp120 mAb are included as nonspecific mAb controls. Presence of each inhibitor indicated by a + sign below. Y-axis indicates % inhibition relative to adhesion in the absence of any inhibitor in three or more independent experiments. Error bars indicate SD. **C)** SPR analysis showing increasing concentrations of ITS03 mAb (2-fold increases from 1.56 nM-25 nM) binding to an HIV cV2 92TH023 coated surface. The parameters of binding kinetics are listed.

We next asked whether SIV V2 mAbs could inhibit α_4_β_7_ adhesion to V2. Five V2 mAbs, ITS03, ITS09, ITS12.01, ITS41, and NCI09 were evaluated using the same strategy outlined above for HIV V2 mAbs (see Fig 3C above). We also included ITS13, a V1 mAb and VRC01 an HIV CD4 binding-site mAb as reagent controls. Mapping studies of these V2 mAbs have been described in detail elsewhere [61] and are summarized in Fig 8A. The epitope for mAb ITS12.01 spans SIV V2 residues 187–197 and falls within S46. It includes the key Asp that is conserved in HIV, SIV and MAdCAM IgSF domain 1. However, ITS12.01 did not block α_4_β_7_ adhesion to SIVmac766 gp120 (Fig 8B). Instead, ITS03 and NCI09, which recognize residues NH_2_-terminal to the ITS12.01 epitope inhibited adhesion most efficiently (>90%). ITS09 and ITS41 also inhibited adhesion, but to a lesser extent, while NCI05, which maps to a region overlapping the ITS09 epitope failed to inhibit adhesion in a detectable way. Of note, the epitopes for both ITS03 and NCI09 do not include the canonical Leu-Asp binding site (Ala^192^-Asp^193^ in SIV) that is conserved in HIV V2 and MAdCAM (Fig 8A). Both of these mAbs do however overlap with the corresponding region of HIV V2, that includes the epitopes for CH58 and CAP228-16H, both of which inhibit α_4_β_7_ adhesion to HIV V2. In summary, a 15-amino acid linear peptide derived from SIV V2 (AA 183–197) inhibits SIV gp120 binding to α_4_β_7_, and SIV mAbs whose epitopes overlap this peptide also inhibit α_4_β_7_ adhesion.

One potential explanation for the conservation of α_4_β_7_ -reactivity between HIV and SIV, despite the divergent V2 sequences represented by peptides H43 and S46 (< 50% sequence similarity) is that the secondary structures of these two peptides share common features [62]. To address this possibility, we screened SIV mAbs for cross-reactivity with HIV V2 and found that ITS03, one of the SIV mAbs that blocked α_4_β_7_ binding most effectively, reacted with relatively high-affinity to HIV cV2 92TH023 (*K*_*D*_ (nM)) 0.105) (Fig 8C). This cross-reactivity is consistent with shared secondary structure between the α_4_β_7_ -binding epitopes localized within the V2 regions of HIV and SIV gp120.

### Macaques treated with an anti-α_4_β_7_ mAb produce anti-V2 antibodies that block α_4_β_7_-adhesion

**Fig 9:**
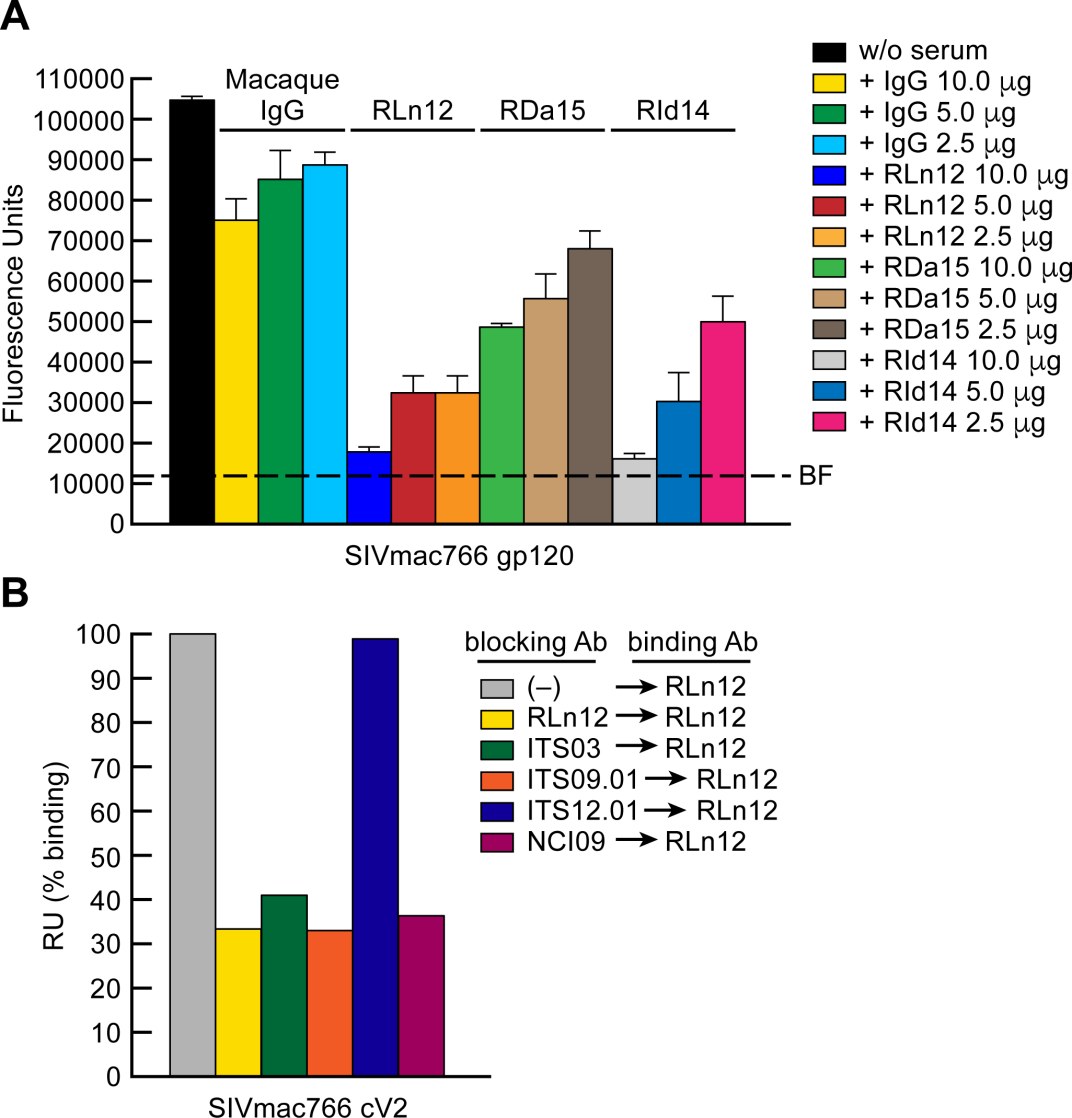
Inhibition of α_4_β_7_ adhesion by serum Ig from macaques treated with ART and Rh-anti-α_4_β_7_. **A)** Adhesion of RPMI8866 cells to the DG form of SIVmac766 gp120 in the absence or presence of 10.0, 5.0, and 2.5 μg of serum Ig from a healthy macaque or three SIV infected macaques, RLn12, RDa15, RId14 treated with ART and anti-α_4_β_7_ mAb. Adhesion was determined at OD_590nm_ and listed as fluorescence units (y-axis). Each condition is run in triplicate. Error bars indicate SEM. These results are representative of three independent experiments. **B)** SPR analysis of serum Ig from SIV infected macaque RLn12 to SIVmac766 cV2 peptide in the absence or presence of pre-bound (blocking) SIV mAbs, ITS03, ITS09.01, ITS12.01 or NCI09, as indicated. Binding is expressed as percent binding RU at 200 seconds after injection of the analyte, relative to binding in the absence of Ig, defined as 100% (y-axis).

SIV infected macaques treated with a combination of ART and a recombinant rhesus anti α_4_β_7_ -mAb (Rh-anti-α_4_β_7_) were able to durably control viremia at relatively low levels following treatment interruption [37]. Although these animals failed to mount neutralizing antibody responses, we noted that following withdrawal of ART, 8/8 generated anti-V2 antibody responses, while only 3/7 control animals administered normal IgG and ART, generated similar responses. Mapping studies indicated that this response was strongly focused on the region of V2 corresponding to S43, the region of V2 that includes the NCI09 and ITS03 epitopes. These V2 responses persisted for at least 50 weeks, despite low plasma viremia [37]. Because NCI09 and ITS03 block adhesion to gp120, we asked whether these polyclonal antibody responses included antibodies that could also inhibit α_4_β_7_ adhesion to gp120. Serum IgG was purified from three ART + anti- α_4_β_7_ mAb -treated animals, RLn12, RDa15, and RId14, by protein G affinity chromatography, and along with normal rhesus macaque IgG was evaluated in an α_4_β_7_ adhesion assay. While normal rhesus IgG showed minimal inhibition of α_4_β_7_-adhesion to SIVmac766 gp120, the sera from all three ART + α_4_β_7_ treated macaques inhibited adhesion in a dose dependent manner (Fig 9A). We then asked whether these sera contained antibodies with specificities similar to the SIV V2 mAbs (described above in Fig 8) that inhibited α_4_β_7_ adhesion to gp120. Using an SPR -based assay, SIVmac766 gp120 coated surfaces were preincubated with SIV V2 mAbs ITS03, ITS09.01, ITS12.01 or NCI09. A surface preincubated with RLn12 serum served as a positive control. Surfaces were then reacted with RLn12 serum. As expected, pre-bound RLn12 serum inhibited RLn12 binding (~70% reduction) (Fig 9B). ITS03, ITS09.01 and NCI09 -mediated similar levels of inhibition. ITS12.01, which showed minimal inhibition of α_4_β_7_ adhesion (Fig 8B), failed to inhibit RLn12 binding (Fig 9B). We conclude that antibody responses generated in SIV infected macaques treated with ART and Rh-anti-α_4_β_7_ included V2 antibodies that target the α_4_β_7_ binding epitope of SIV V2.

## Discussion

In this study we report that the way in which α_4_β_7_ interacts with the V2 region of gp120 shares key features with the interaction between α_4_β_7_ and its natural ligand, MAdCAM. This apparent mimicry may have important implications in HIV pathogenesis, particularly in regard to the role of the gut in the development of HIV disease. It may also impact anti-V2 loop immune responses in both infected and vaccinated subjects. One consequence of this mimicry is that antagonists developed to treat IBD interfere with V2-α_4_β_7_ interactions. The V2 domains of HIV and SIV gp120 vary in both length and sequence identity. Yet we find that V2s from three subtypes of HIV, as well as a V2 from SIV retain the capacity to bind to α_4_β_7,_ suggesting that this interaction is a general property across HIV subtypes. Other studies failed to detect a specific interaction between α_4_β_7_ and gp120 [13, 14]. An explanation for this discrepancy likely reflects two variables. First, as we and others have shown, the addition of glycans can reduce the interaction between recombinant gp120 with α_4_β_7_ [16, 58]. It is likely that excess amounts of complex carbohydrate and sialic acid moieties that are added to gp120s expressed in cell lines contribute to this inhibitory effect. Of note, we demonstrated that highly purified (>95%) cyclic V2 loop peptides that lack glycans bind α_4_β_7_ with high affinity in both SPR -based assays and in a cell -based adhesion assay. Removal of glycans in order to observe α_4_β_7_ reactivity in not an absolute requirement insofar as we were able to capture virions derived from primary CD4^+^ T cells with α_4_β_7_ coated nanoparticles. The second variable that may influence the sensitivity of α_4_β_7_ binding assays involves the expression level, and state of α_4_β_7_ on cell surfaces. Many integrins rely on complex avidity effects and clustering in order to engage ligands. It is likely that the surface density of α_4_β_7_ plays a key role in its interaction with gp120.

The specific affinity of α_4_β_7_ for gp120 is comparable to that of MAdCAM. Among the 24 integrins expressed in humans, the α_4_β_7_ heterodimer is distinct in its ability to mediate both lymphocyte rolling, and firm adhesion, which reflects the highly specialized nature of MAdCAM-α_4_β_7_ interactions. These two functions are achieved by dynamic changes in the overall structure of the heterodimer. In this regard, it is notable that the V2 region of gp120, despite its variable sequence, is able to mimic the binding of α_4_β_7_ to MAdCAM. The evidence for mimicry comes from two observations. First, the manner in which V2 depends upon divalent cations to engage α_4_β_7_ tracks closely with the way cations are used by MAdCAM. To bind α_4_β_7_, MAdCAM utilizes divalent cations. A Mg^++^ ion sits in the MIDAS of β_7_ and coordinates with an Asp in the ligand (Asp^42^ in MAdCAM). Mn^++^ occupies the MIDAS in a more stable way, so that replacing Mg^++^ with Mn^++^ results in an apparent increase in affinity. This pattern holds for gp120 V2, indicating that V2 appears to interact with the different conformations of α_4_β_7_ in the same way that MAdCAM does. Importantly, the conformational state (inactive, intermediate or active) of α_4_β_7_ is responsive to both intracellular and external cues that are linked to cellular signals generated during inflammatory responses [63, 64]. The ability of V2 to discriminate between different forms of α_4_β_7_ provides a mechanism to distinguish between different subsets of lymphocytes, including those with high potential to home to GALT. Given the propensity of HIV to replicate in GALT, it is tempting to link the preferential infection and depletion of α_4_β_7_^high^ CD4^+^ T cells in the very early stages of infection [8, 32, 65], with V2-α_4_β_7_ interactions. However, such a link has not yet been established.

The second line of evidence that supports the proposition that V2 mimics the binding characteristics of MAdCAM comes from our demonstration that α_4_β_7_ antagonists that were developed to block binding to MAdCAM, also block binding to V2. The incidence and prevalence of IBD is increasing throughout the world [66], and consequently there has been a concerted effort to develop effective treatments, including drugs that target α_4_β_7_. To this end, detailed structural characterizations of both MAdCAM and α_4_β_7_ have been employed in the rational design of small molecule LDV mimetics [67]. These mimetics bind with precision to the MAdCAM binding site on α_4_β_7_, which lies within a ~ 40 Å long, 10 Å deep groove formed by the α_4_-β_7_ interface (Fig 2A) [23]. By showing that one of these mimetics competes with V2 we conclude that the aliphatic amino acid-Asp motifs conserved in both HIV and SIV fit into this groove and engage α_4_β_7_ in a way that, at least partially, mimics the Leu^41^-Asp^42^ encoded in the MAdCAM CC' loop of IgSF domain 1 (Figs 2B and 6G) [52, 68]. These results suggest that the carboxy-terminus of V2 and this IgSF domain 1 CC' loop can adopt similar conformations. Evidence for the conserved nature of this structure comes from our observation that one SIV V2 mAb, ITS03, whose epitope maps close to the α_4_β_7_ binding site, blocks α_4_β_7_ adhesion to V2 and also cross-reacts with an HIV subtype A/E V2. This raises the intriguing possibility that, with additional screening, one might identify a V2 mAb that cross-reacts with MAdCAM. Indeed, other regions of the HIV envelope have shown to mimic “self” epitopes [69].

Vedolizumab is a unique α_4_β_7_ antagonist. It binds exclusively to the SDL of β_7_ in the context of α_4_β_7_; however, structural constraints preclude it from binding to α_E_β_7_ [23]. It inhibits V2-α_4_β_7_ adhesion, which further supports the idea that V2 mimics MAdCAM. However, the mechanism by which vedolizumab, and its parent mAb, Act-1 [70], interfere with MAdCAM binding is less well defined than that for LDV mimetics. The key to understanding this mechanism is understanding the role of MAdCAM IgSF domain 2. Mutagenesis or deletion of the charged C'-E loop of MAdCAM IgSF domain 2 abrogates α_4_β_7_ binding [49, 50]. Docking experiments carried out in silico indicate that this loop comes in close proximity to the β_7_ SDL and may contact α_4_β_7_ directly (Fig 2A and 2B). By binding to the SDL, vedolizumab is likely to prevent direct interactions with the DE loop and/or sterically interfere with interactions between IgSF domain 1 and the binding groove at the interface between α_4_ and β_7_. As such, the α_4_β_7_ binding footprint on MAdCAM spans two IgSF domains, and interactions with two loops that are separated by ~18-36Å that are both involved in binding to α_4_β_7_. This raises a question regarding our demonstration that vedolizumab blocks adhesion between V2 and α_4_β_7_. We show that a limited region within a ~40 AA cV2 peptide appears to engage the binding groove. The limited size of these cV2 peptides suggests that simultaneous interactions between these peptides and both the binding groove and the SDL are unlikely. Thus, the mechanism by which vedolizumab blocks V2 binding remains unclear and requires further investigation.

Although the evidence for molecular mimicry outlined above is strong, the extent of this mimicry is likely to be limited. Because the interaction between MAdCAM and α_4_β_7_ facilitates both rolling and firm adhesion of lymphocytes along the endothelium, it encompasses a high degree of complexity that may not be entirely reflected in the interaction between V2 and α_4_β_7_. Moreover, the region of V2 involved in binding to α_4_β_7_ is variable which makes it unlikely that it could retain the functional complexity inherent in the interactions that occur between MAdCAM and α_4_β_7_.

We found that certain weakly neutralizing V2 mAbs elicited from both infection (CAP228-16H) and vaccination (CH58) could inhibit V2 -mediated adhesion to α_4_β_7_. In this regard, there is a growing interest among HIV vaccine researchers regarding the potential utility of weakly neutralizing, or “functional” antibodies. This stems in large measure from the results of the RV144 Phase III vaccine trial in Thailand, where risk of acquisition was found to correlate inversely with weakly neutralizing V1/V2 antibodies [71–75]. Subsequently, a molecular sieve analysis of viral quasi-species in vaccinated individuals who became infected showed that residues at positions 169 and 181 within the V2 region were subject to immune pressure around the time of infection (Fig 3B) [76]. These residues fall around the binding epitopes for both CH58 and α_4_β_7_. Follow-up studies suggested that antibody effector functions might contribute to the observed RV144 risk-reduction [77, 78]. Our finding that mAb CH58, which was derived from an RV144 vaccine recipient, block V2 -mediated adhesion to α_4_β_7_ raise the possibility that antibody activities distinct from both neutralization and Fc -mediated effector functions might contribute to the efficacy of HIV vaccines. Supporting this concept, we found that mAb NCI09, which was derived from a macaque administered an SIVmac251-based vaccine designed to mimic the RV144 vaccine [79], also blocked V2 -mediated adhesion to α_4_β_7_. As in RV144, reduced risk of infection in animals administered this vaccine was correlated with weakly neutralizing V2 antibodies [79]. A more complete description of this antibody and its activities are described elsewhere (Franchini et al., in preparation).

How weakly neutralizing antibodies that block V2 -mediated adhesion to α_4_β_7_ might contribute to reduced risk of infection is unknown. We previously reported that the V2 region of gp120 can deliver cellular signals through α_4_β_7_ [9]. In this regard, integrins including α_4_β_7_ are key components in integrin associated complexes that are able to modulate biochemical pathways and reorganize both cell-surface receptors and the actin-cytoskeleton [63, 80]. In addition, α_4_β_7_ can deliver costimulatory signals to CD4^+^ T cells that impact cell activation, proliferation and apoptosis [81, 82]. We recently reported that MAdCAM -mediated costimulation supports HIV replication in α_4_β_7_^high^ CD4^+^ T cells [19]. Whether V2 signaling through α_4_β_7_ can similarly facilitate HIV replication requires additional investigation. Such information will help determine whether this type of signaling could facilitate HIV transmission/replication and whether antibodies that interfere with this signal can reduce the risk of infection.

The nature of the epitopes recognized by mAbs that inhibit the interaction between V2 and α_4_β_7_ should help us identify the context in which these interactions occur in vivo. It is noteworthy that these weakly neutralizing mAbs recognize an epitope that is structurally distinct from the β- strand that is presented on the closed BG505 SOSIP trimer. As such, broadly neutralizing mAbs that do recognize epitopes on the closed BG505 SOSIP trimer are unlikely to be effective inhibitors of V2-α_4_β_7_ interactions. Instead, we show that a unique α_4_β_7_ -reactive conformation of V2 is formed when it is relieved from constraints mediated by other domains of gp120/41.

Although the context in which V2 engages α_4_β_7_ remains to be determined, evidence is accumulating that α_4_β_7_ -expressing cells play an important role in the early stages of HIV/SIV infection. The recent demonstration by Sivro and colleagues that α_4_β_7_^high^ CD4^+^ T cells are preferentially depleted from gut tissues as early as Fiebig I/II provides strong evidence that these cells serve as prime early targets for infection following transmission, an observation that is consistent with our demonstration that an α_4_β_7_ mAb protects macaques from vaginal challenge [36]. However, we cannot exclude the possibility that α_4_β_7_ plays a broader role in HIV pathogenesis. When we combined this same α_4_β_7_ mAb with ART, SIV infected animals were able to control viremia in a sustained way [37]. In trying to identify the underlying mechanism of control we reported, among other findings, that each of the controlling animals generated anti-V2 specific antibody responses that mapped to the region of SIV V2 that corresponds to the CH58 epitope in HIV V2. In this report, we show that these serum V2 antibodies can block V2 -mediated adhesion to α_4_β_7_. This finding underscores the need to further explore the role of V2 α_4_β_7_ interactions in HIV pathogenesis.

In conclusion, the way that the V2 region of gp120 engages α_4_β_7_ shares key features with the way that MAdCAM, a receptor expressed primarily in gut tissues, engages α_4_β_7_. One consequence of this apparent mimicry is that antagonists developed to treat IBD by interfering with the interaction between MAdCAM and α_4_β_7_ also interfere with V2-α_4_β_7_ interactions. The nature of the epitope in V2 that engages α_4_β_7_ appears to involve a structure that is not present in a recombinant SOSIP trimer designed to mimic the closed trimeric spike that is the target of most broadly neutralizing antibodies. Antibodies that target this epitope and block V2-α_4_β_7_ interactions are not themselves broadly neutralizing, although the structure that they recognize is conserved across clades and is present in SIV V2. These findings suggest that mimicry of MAdCAM-α_4_β_7_ interactions by V2 may influence early events in HIV infection and replication in GALT.

## Materials and methods

### Ethics statement

Generation of mAb Mk16C2 was approved and carried out under animal use protocol A-1896 by the Institutional Animal Care Committee (IACUC) of the University of Massachusetts Medical School. The University of Massachusetts Medical School is fully accredited by Association for Assessment and Accreditation of Laboratory Animal Care (AAALAC) and has an Animal Welfare Assurance on file with the Office of Laboratory Animal Welfare (OLAW). Assurance number: A-3306-01.

### Cell line and reagents

RPMI8866 cells, a human B lymphoma cell line that constitutively expresses α_4_β_7_ was purchased from Sigma-Aldrich. Cells were maintained in RPMI-1640 (Lonza) containing 10% heat inactivated fetal bovine serum (Gibco), 2% penicillin/streptomycin/glutamine (Gemini Bio-Products) and 0.1% retinoic acid (RA). Cells were cultured for a minimum of 7 days prior to use in adhesion assays. RA was obtained from Sigma Chemical. CH58, human mAb isolated from RV144 vaccinated individuals, and VRC01 were provided by the NIAID AIDS reagent program. CAP228-16H mAb was generated in the laboratory of Dr. Lynn Morris (CAPRISA) [57]. The 830A mAb was provided by Dr. Susan Zolla-Pazner (Mt. Sinai Medical School) [42]. Rabbit Mk16C2 mAb (provided by Dr. Shan Lu, University of Massachusetts Medical School) was isolated from a rabbit that received a gp120-JRFL DNA prime- protein boost immunization, using V2-peptide specific single B cell sorting, and produced by transfection of cloned Ig genes in 293F cells at the University of Massachusetts Medical School [83]. The SIV mAbs ITS03, ITS09.01, ITS12.01, ITS13, and ITS41 were provided by Dr. Rosemarie Mason, NIAID VRC [61]. NCI09 was provided by Genoveffa Franchini and produced in the laboratory of Rosemarie Mason in a manner identical to mAbs mAbs ITS03, ITS09.01, ITS12.01, ITS13, and ITS41 [61]. Rhesus macaque serum from animals RLn12, RDa15, RId14 was provided by Dr. Aftab Ansari (Emory University) [37], was obtained after 72 weeks post-infection, and more than 40 weeks after the last infusion of anti-α_4_β_7_ mAb. Serum was purified by protein G column chromatography and dialyzed into HBS. Cyclic V2 peptides with > 90% purity and having an amino-terminal biotin, derived from 92TH023, BG505 and C06980v0c22 were supplied by JPT Peptide Technologies. Linear HIV and SIV 15 amino acid peptides were obtained from the NIAID AIDS Reagent Repository, or from Biopeptide Co. and supplied at >90% purity. Scaffolds V1/V2 92TH023-1FD6 and V1/V2 92TH023-Tag were constructed, expressed and purified as described elsewhere [42]. CHO cell derived A244 gp120 (Lot 26539–1) was provided by Global Solutions for Infectious Diseases (San Francisco, CA). Purification employed an anti-gD immunoaffinity resin, followed by both cation and anion exchange chromatography steps. Purity was estimated at 97.1%. CHO cell derived SIVmac766 gp120 was provided by Advanced Biotechnologies Laboratories. BG505 SOISP trimer was generously provided by Dr. Paolo Lusso, LIR/NIAID. Vedolizumab was obtained from the NIH Clinical Center Pharmacy Department. Human integrin α_4_ mAb 2B4, MAdCAM-Ig, soluble α_4_β_7_ and α_4_β_1_ were obtained from R&D Systems. The LDV mimetic ELN-475772 was provided by ELAN Pharmaceuticals [84]. Conjugation of HRP to mAbs was carried out using a LYNX HRP conjugation kit obtained from Bio-Rad, using the manufacturer’s instructions.

### Deglycosylation of purified gp120 and gp140 protein

Prior to use purified gp120 and gp140 proteins utilized in the α_4_β_7_ adhesion assays were first treated with a deglycosylation protocol [58]. Purified proteins were treated with 500U of PNGase F (NEB) per 20 μg of protein under non-denaturing conditions (1X GlycoBuffer 2 (NEB), 5mM DTT, and water) at 37°C for 3 hours.

### Surface plasmon resonance analysis of α_4_β_7_ or anti-V2 loop mAb binding to Hu-MAdCAM, gp120 or cyclic V2 peptides

Experiments were performed using a Biacore 3000 instrument (GE Life Sciences) using CM4 or CM5 sensor chips. The data were evaluated using BIAevaluation 4.1 software (GE Life Sciences). The chip surface was activated by injecting 35 μl of a 1:1 mixture of 0.05 M *N*-hydroxysuccinimide and 0.2 M *N*-ethyl-*N*-(dimethylaminopropyl) carbodiimide at 5 μl/min. NeutrAvidin, HIV gp120 or Hu-MAdCAM-Ig (R&D Systems) at concentrations of 5 μg/ml in 10mM NaOAc, pH 4.5, were immobilized to approximately 750 resonance units (RU). After the proteins were immobilized to the desired densities, unreacted sites on each surface were blocked with 35 μl of 1 M Tris-HCl (pH 8.0). Biotinylated cyclic V2 peptides (1 μg/ml in 20 mM Tris-HCl, pH 8.0) were bound to the NeutrAvidin surfaces to densities of approximately 250–300 RU. One surface was activated and blocked without ligand to act as a control surface for non-specific binding of the soluble ligand. Any binding was subsequently subtracted from the remaining surfaces. Running buffer was HBS (pH 7.4), 0.01 mM CaCl_2_, either 1 mM MgCl_2_ or MnCl_2_, 0.005% Tween-20, 0.05% soluble carboxymethyl-dextran. Binding experiments were carried out at a flow rate of 25 μl/min at 25°C. After a 2 min injection, the surface was washed for an additional 2 min in running buffer to follow dissociation of the bound ligand from the surface. The surfaces were regenerated by multiple injections of 4.5 M MgCl_2_ at a flow rate of 100 μl/min. Inhibition of α_4_β_7_ or anti-V2 loop antibodies by linear V2-loop peptides was carried out by pre-incubating the proteins with the peptides in running buffer at the indicated concentrations for 2 hours at room temperature prior to passing them over the prepared surfaces as described above.

### Kinetic analysis of anti-V2 loop mAbs binding to cyclic V2 peptides

Antibodies were diluted to the indicated concentrations in running buffer prior to being sequentially passed over the surface bound cyclic peptides as described above. The resulting sensorgram series were analyzed using the BiaEvaluation 4.1 software (GE Life Sciences) and fitted using a 1:1 Langmuir binding model to determine the kinetic rate and affinity constants.

### Crystallization, data collection, structure determination and refinement of Mk16C2 in complex with V2 peptide

The Fab fragment of rabbit mAb Mk16C2 (provided by Dr. Shan Lu, Univ. of Massachusetts Medical School) was prepared by papain digestion as described (PMID: 19913488 and 20622876). Briefly, the IgG molecule was mixed with papain (Worthington, Lakewood, NJ) at a 20:1 molar ratio in 100 mM Tris (pH 6.8) with 1 mM cysteine hydrochloride and 4 mM EDTA. The mixture was incubated for 1 hour at 37°C and the reaction was stopped by 10 mM iodoacetamide. The Fab fragment was separated from the Fc fragment and the undigested IgG by a protein A column and further purified by size exclusion chromatography. The Fab fragment was then concentrated to about 10 mg/ml for crystallization.

The 15mer V2_ConB_ peptide (RDKVQKEYALFYKLD) was dissolved in water and mixed with Fab of rabbit mAb Mk16C2 in excess at a 10:1 molar ratio. Crystallizations conditions were screened and optimized using the vapor diffusion hanging drop method. Well-diffracted crystals of Mk16C2 Fab/V2_ConB_ complex were obtained with a well solution of 23% polyethylene glycol 3350, 0.2 M LiCL, 0.1 M 2-ethanesulfonic acid (MES) pH 6.5, and soaked briefly in the crystallization solution with an additional 20% glycerol before being flash frozen in liquid nitrogen. X-ray diffraction data sets were collected at the synchrotron beamline 14–1 of Stanford Synchrotron Radiation Lightsource (SSRL) of Stanford Linear Accelerator Center (SLAC) National Accelerator Laboratory. All data sets were processed using the XDS (PMID: 20124692), and structures determined by molecular replacement using another rabbit mAb R56 Fab structure (PDB ID 4JO1) as the initial model. Cycles of refinement for each model were carried out in COOT (PMID: 15572765) and Phenix (PMID: 20124702). Final structural analyses were carried out using ICM and figures were generated using PyMOL (pymol.org) and ICM (www.molsoft.com). The antigen-antibody interactions described in Fig 3A are calculated by PDBePISA (EMBL-EBI). Coordinate and structure factor of the complex have been deposited in the Protein Data Bank under PDB ID 6CEZ.

### α_4_β_7_ adhesion to MAdCAM-1, HIV gp120, HIV cyclic V2 peptide and SIV gp120

The binding of α_4_β_7_ expressed by the RPMI8866 cell line to MAdCAM, HIV A244 gp120, HIV cyclic V2 peptides, and SIVmac766 gp120 gp120 in the absence or presence of vedolizumab or 2B4 or ELN-475772 to MAdCAM, HIV A244 gp120 were analyzed by an adhesion assay (adapted from KK Peachman et al., [11] (S2A Fig). This assay was modified by culturing RPMI8866 cells in media containing 1μM RA for at least 7 days prior to use in adhesion assays. Inclusion of RA increases adhesion to cV2 peptides, gp120 and MAdCAM (S2C Fig). Briefly, triplicate wells of a 96-well flat-bottom polypropylene plate (Greiner Bio-One) were coated overnight at 4°C with 100 μl of 2 μg/ml of MAdCAM-1 (R & D Systems) or 100 μl of 2 μg/ml NeutrAvidin or 100 μl of 0.5–2.0 μg of deglycosylated SIV and HIV gp120 diluted in 50mM bicarbonate buffer, pH 9.6. The NeutrAvidin-coated plates were then incubated with biotinylated cyclic V2 peptides (5 μg/ml in bicarbonate buffer) for 1 hour at 37°C. The solution from the plates was discarded and the plates were then blocked with blocking buffer (25mM Tris, 2.7mM potassium chloride, 150 mM sodium chloride, 0.5% BSA, 4mM manganese chloride, pH 7.2) for 1 hour at 37°C. The solution was discarded and plates were manually washed 4 times with blocking buffer. After blocking and washing the plate, RPMI8866 cells in a volume of 50 μl/well were pre-incubated for 40 min at 37°C with sample buffer in the absence or presence of 10 μg/ml of vedolizumab (α_4_β_7_ mAb) or 2B4 (α_4_ mAb) or ELN-475772 (α_4_β_7_/α_4_β_1_ dual inhibitor). Plates were then incubated with 50 μl/well of 2x10^5^ RPMI8866 cells at 37°C (5% CO_2_) for 1 hour, washed 5 times with PBS followed by the addition of 100 μl of RPMI-1640 containing 1% FBS, 1% pen/strep/glutamine, 25mM HEPES with 10 μl/well of AlamarBlue dye. Fluorescence (excitation 560 nm and emission 590 nm) was measured immediately after the addition of the AlamarBlue dye for 8 hours.

### Capture of 92TH023 virions by MNPs coated with gp120 mAbs or α_4_β_7_

92TH023 virions were captured with 15 nm magnetic nanoparticles (MNPs) coupled to α_4_β_7_ or 2G12, CH58, PG9, and 830A mAbs as previously described [85]. Briefly, carboxyl-terminated iron oxide nanoparticles (Ocean Nanotech, San Diego) via two step carbodiimide reaction were coated with 1mg of anti-gp120 mAbs or recombinant soluble α_4_β_7_ according to manufacturer’s protocol. Virus preparations were derived from primary CD4^+^ T cells infected with an infectious molecular clone derived from 92TH023, using standard conditions. To capture virions, MNPs coated with mAbs (3.9 x10^12^) in 60μl were incubated with viral preparation (33 ng/ml based on p24 content) for 1hour at 37°C. Captured virions were separated on MACS magnetic columns attached to an OctoMacs magnet (Miltenyi Biotech) washed 4 times with 600 μl wash buffer (0.5% bovine serum albumin, 2mM EDTA in PBS), eluted in 200 μl PBS and analyzed on Luminex X200 for p24 content using a dynamic immunofluorescent cytometric bead assay [60].

In experiments with α_4_β_7_-MNPs, virions were incubated in the absence or presence of increasing amounts (1.25nM, 12.5nM, 125nM) of the α_4_β_7_ inhibitor ELN-475772. Incubation and washing were performed in complete medium with 1mM MnCl_2_.

### Inhibition of α_4_β_7_ adhesion to HIV gp120, HIV cyclic V2 peptides and SIV gp120 by V2 monoclonal antibodies and polyclonal sera

Triplicate wells of a 96-well flat-bottom polypropylene plate (Greiner Bio-One) were coated with biotinylated cyclic V2 peptides, deglycosylated SIV and HIV gp120 as described above. After blocking and washing, plates were incubated with 20–100 μg/ml of the designated anti-V2 mAbs or 2.5–10.0 μg of protein G purified IgG from sera drawn from SIV infected rhesus macaques (RLn12, RDa15, RId14 (provided by Dr. Aftab Ansari, Emory University School of Medicine)) in sample buffer (25 mM Tris, 2.7 mM KCl, 150 mM NaCl, 4 mM manganese chloride, 1% fetal bovine sera, pH 7.2) for 1 hour at 37°C. RPMI8866 cells were pre-incubated for 40 min at 37°C with sample buffer. Plates were then incubated with 50 μl/well of 2x10^5^ cells at 37°C (5% CO_2_) for 1 hour, washed 5 times with PBS followed by the addition of 100 μl of RPMI-1640 containing 1% FBS, 1% pen/strep/glutamine, 25mM HEPES with 10 μl/well of AlamarBlue dye (Invitrogen). Following the addition of AlamarBlue dye (excitation 560 nm and emission 590 nm) fluorescence was measured for a period of 8 hours.

### α_4_β_7_ adhesion to constrained and unconstrained V1/V2 92TH023 scaffolds

92TH023 V1/V2 was cloned into both 1FD6 (constrained) and tag (unconstrained) scaffolds. Plates were then coated with 0.5, 1.0, and 2.0 μg of either V1/V2 92TH023 1FD6 or V1/V2 92TH023 tag scaffolds followed by addition of RPMI8866 cells. The plates were washed and 100 μl of media and 10 μl of AlamarBlue dye was added to each well as described above. Fluorescence (excitation 560 nm and emission 590 nm) was measured immediately after the addition of the AlamarBlue dye.

### Binding of cyclic V2 peptide to V2 monoclonal antibodies by ELISA

Briefly, Corning Costar 96-Well plates were coated with NeutrAvidin at 4°C overnight. Wells were washed six times with wash buffer (water, 1 mM MnCl_2_, and 1X plate wash buffer) using Microplate Washer ELx50, (Bio Tek Instruments), and then blocked with blocking buffer (HBS, 5% bovine serum albumin) for 1 hour at room temperature. Plates were coated with 1 μg/ml biotinylated cyclic V2 peptides in binding buffer (HBS, 1 mM MnCl_2_) for 1 hour at room temperature. The plates were washed six times with wash buffer and HRP-conjugated anti-V2 mAb was added to wells for 1 hour at room temperature. After washing, 100 μl/well of substrate was added for 10 min to develop color at room temperature in the dark. Plates were read at OD_450_ nm using an EnSpire Multimode Plate Reader (PerkinElmer).

### CH58 mAb and 830A mAb competition ELISA

10, 20, 50, 80 and 160 ng of the CH58 mAbs in binding buffer (HBS, 1 mM MnCl_2_) were incubated with 50 ng of HRP-conjugated 830A mAb and then added to a plate coated with 100 ng of cV2 92TH023. Substrate was added using conditions specified by the manufacturer. Plates were read at OD_450_ nm using an EnSpire Multimode Plate Reader, PerkinElmer.

### Immunofluorescence staining

RPMI8866 cell line cultured in the presence or absence of RA were seeded at 1×10^5^/well in Poly-d-lysine coated glass bottom dishes (MatTek) with cover glasses and incubated overnight at 37°C. The cells were fixed with 2% paraformaldehyde (PFA), blocked with 1% BSA, and stained with an anti-β_7_ PE mAb (BD Biosciences) or an IgG2a-PE isotype control mAb (R & D Systems). Stained RPMI8866 cells were microscopically analyzed using a Leica SP8 confocal microscope (Leica Microsystem, Inc.) and images were processed with Leica LAS AF software (Leica Microsystem, Inc.) and Imaris software v.9.0.1 64x (Bitplane AG).

## Supporting information

S1 Fig Binding of HIV V2 specific mAbs to the V2 domain of 92TH023. **A)** List of the HIV and SIV V2 domain sequences used in this study, along with GenBank accession numbers, and amino acid numbering derived from the corresponding full-length gp160. **B)** ELISA binding of HIV V2 specific mAbs CH58, 830A, CAP228-16H and Mk16C2 to wells coated with NeutrAvidin and a cyclic V2 peptide derived from 92TH023 gp120. PBS was used as negative control. Binding measured at OD_450nm_ (y-axis). **C)** ELISA binding of unlabeled CH58 competing with HRP-830A binding to cV2 92TH023. Concentration of CH58 listed on the x-axis. Binding measured at OD_450nm_ (y-axis).
(TIF)

S2 Fig The gp120 V2 adhesion assay and effect of RA on α_4_β_7_ adhesion. **A)** Schematic of adhesion assay. α_4_β_7_ -expressing RPMI8866 cells (yellow) were incubated in the absence or presence of anti-α_4_β_7_ or -β_7_ mAbs (Vedolizumab, 2B4) (dark blue) and then added to plates previously coated with the cV2 peptide (purple). Alternatively, cV2 peptides are pre-incubated with anti-V2 specific mAbs (green). Plates were washed and AlamarBlue dye was added to each well. Fluorescence was measured for 8 hours at 1-hour intervals (OD_590nm_). **B)** Expression and distribution of β_7_ on RPMI8866 cells +/- RA, stained with an anti-β_7_ PE mAb or an IgG2a-PE isotype control mAb viewed by confocal microscopy. Upper panels: differential interference contrast (DIC), lower panels: fluorescence (red). **C)** Adhesion of RPMI8866 cells, cultured in the presence (+RA) or absence (-RA) of retinoic acid, to MAdCAM-Ig, or cyclic V2 peptides derived from HIV 92TH023, C06980v0c22, and BG505. Adhesion was determined by OD_590nm_ and listed as fluorescence units (y-axis). Background fluorescence (BF) of RPMI8866 cell adhesion to a blank well is denoted by a dashed line.
(TIF)

S3 Fig α_4_β_7_ adhesion to MAdCAM or cV2 92TH023 under different cation conditions. **A**-**B**) Adhesion of RPMI8866 cells to immobilized MAdCAM or a cV2 92TH023 peptide in the buffers containing a low concentration of divalent cations, or high concentrations of MnCl_2_ or MgCl_2_ as reported in Fig 2F in two additional independent experiments. Adhesion was determined at OD_590nm_ and listed as fluorescence units (y-axis). Conditions are run in triplicate and error bars indicate standard error of the mean (SEM). Background fluorescence (BF) of RPMI8866 cells to blank wells is denoted by a dashed line.
(TIF)

S4 Fig α_4_β_7_ adhesion to A244 gp120 in the absence or presence of V2-specific mAbs. Adhesion of RPMI8866 cells to immobilized deglycosylated A244 gp120 in presence of HIV V2-specific mAbs: CH58, CAP228-16H, and Mk16C2. The LDV mimetic ELN-475772 was included as a specificity control (spec. ctrl) for adhesion to α_4_β_7_. Average adhesion in three or more independent experiments is reported as fold-change in adhesion relative to undeglycosylated A244 gp120 (y-axis). Error bars indicate SD. Significance determined by unpaired t-test (*p <0.05).
(TIF)

S5 Fig α_4_β_7_ adhesion to deglycosylated BG505 SOSIP trimer, A244 gp120, and SIVmac766 gp120. Adhesion of RPMI8866 cells to immobilized DG forms of BG505 SOSIP trimer, A244 gp120, and SIVmac766 gp120 relative to corresponding fully glycosylated forms of each protein expressed as fold-change (y-axis). Results from three independent experiments are shown. Error bars indicate SD. Significance determined by unpaired t-test (*p <0.05).
(TIF)

S6 Fig BG505 SOSIP vs. cV2 BG505 peptide adhesion to α_4_β_7_. Adhesion of RPMI8866 cells to BG505 SOSIP trimer or cV2 BG505. Results from three or more independent experiments are shown and reported as % adhesion relative to cV2 BG505 in the absence of any inhibitor or in the presence of a specific inhibitor. The anti-α_4_ mAb 2B4 which was employed as a specificity control (spec. ctrl) for cV2 BG505, and VRC01 was employed as a nonspecific mAb control for cV2 BG505. Error bars indicate SD. Significance determined by unpaired t-test (***p <0.001 and ****p <0.0001).
(TIF)

S1 Table Surface plasmon resonance detailed binding parameters. (TIF)

S2 Table Mk16C2 structure refinement parameters. (TIF)
