# Supplementary figures and images for: Select gp120 V2 domain specific antibodies derived from HIV and SIV infection and vaccination inhibit gp120 binding to α4β7
Source: PLoS Pathog. 2018 Aug 28;14(8):e1007278. doi: 10.1371/journal.ppat.1007278 (PMC6130882; doi:10.1371/journal.ppat.1007278)

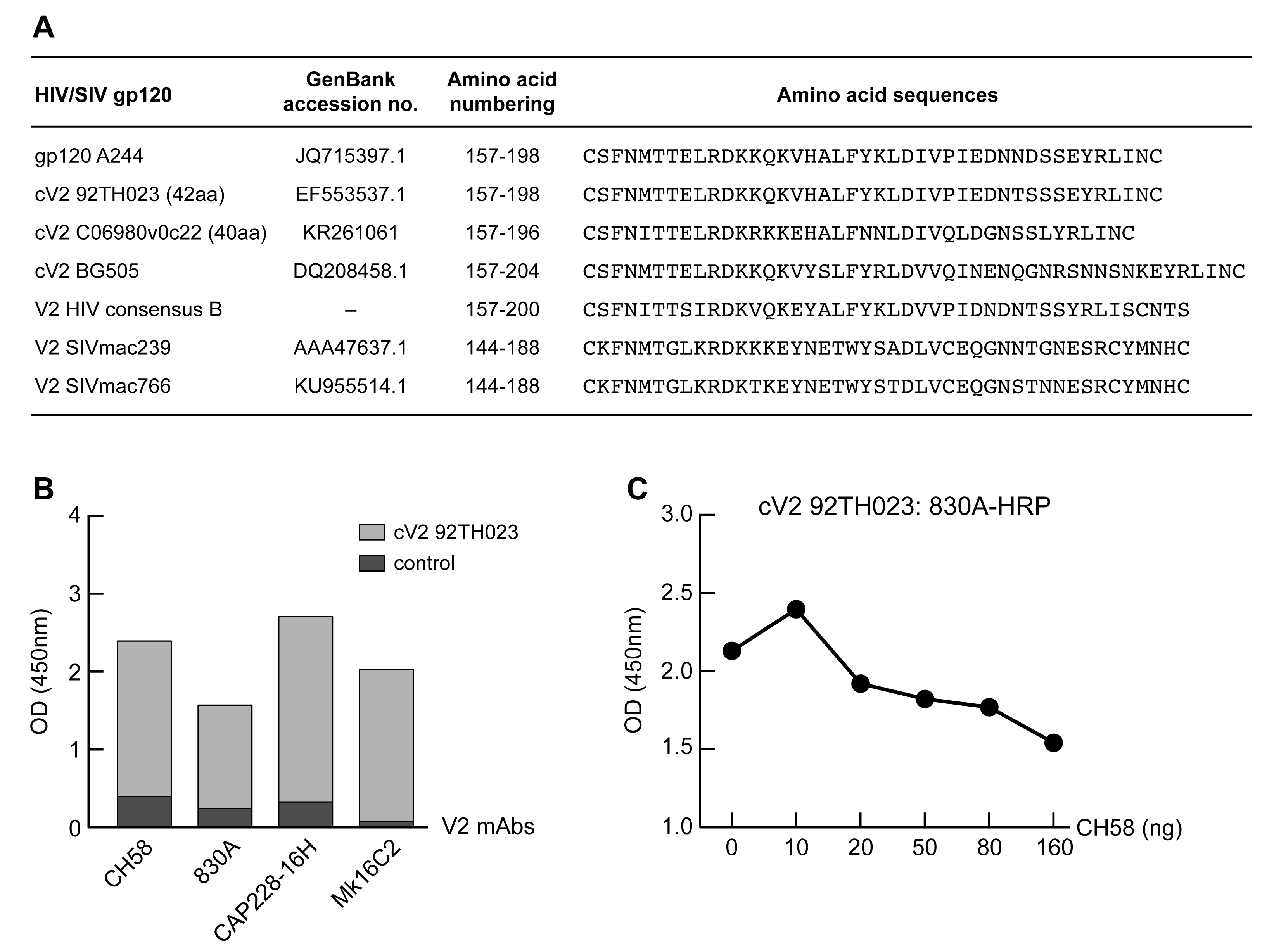

Supplement: S1 Fig — A) List of the HIV and SIV V2 domain sequences used in this study, along with GenBank accession numbers, and amino acid numbering derived from the corresponding full-length gp160. B) ELISA binding of HIV V2 specific mAbs CH58, 830A, CAP228-16H and Mk16C2 to wells coated with NeutrAvidin and a cyclic V2 peptide derived from 92TH023 gp120. PBS was used as negative control. Binding measured at OD450nm (y-axis). C) ELISA binding of unlabeled CH58 competing with HRP-830A binding to cV2 92TH023. Concentration of CH58 listed on the x-axis. Binding measured at OD450nm (y-axis). (TIF) [file ppat.1007278.s001.tif]

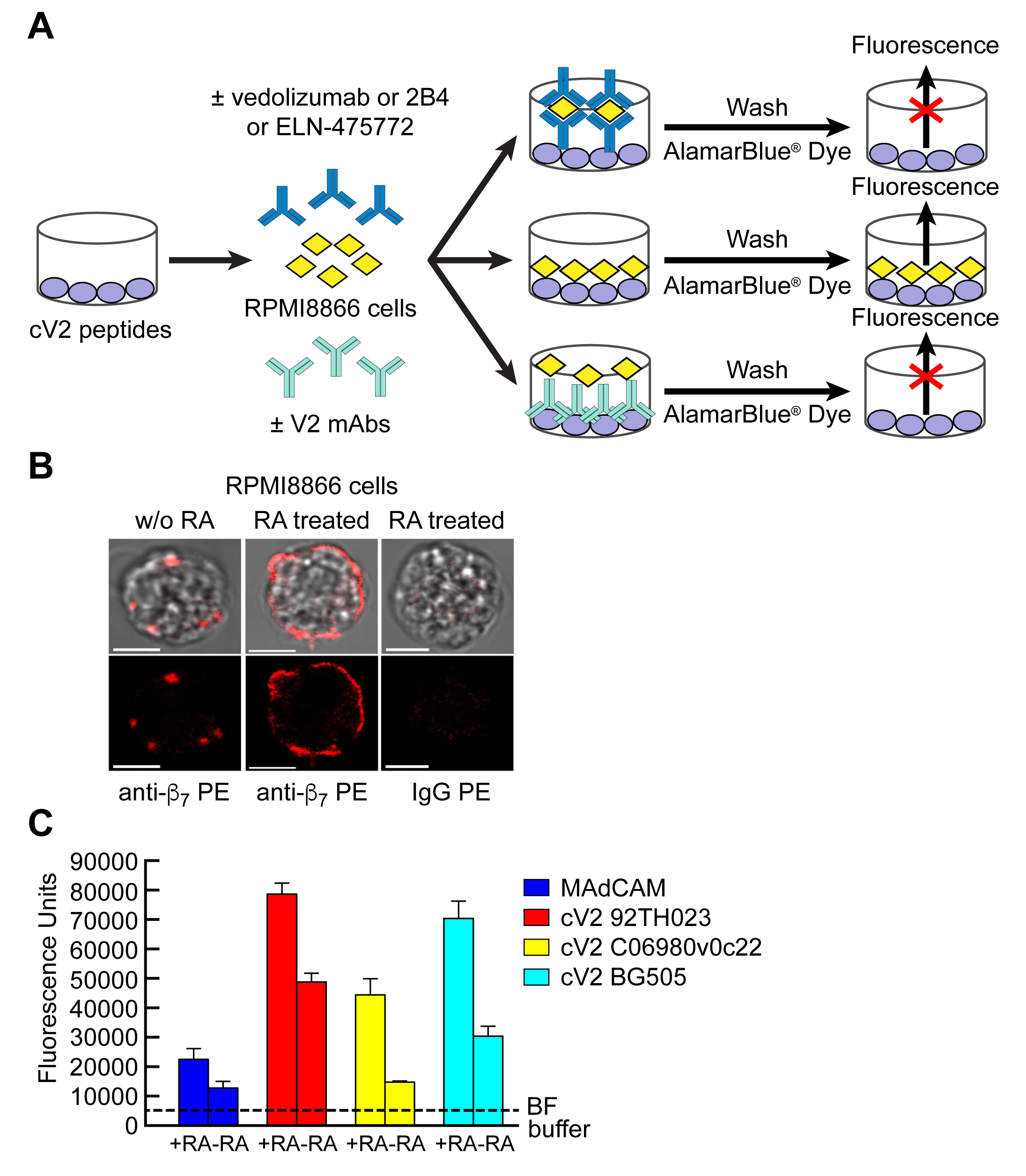

Supplement: S2 Fig — A) Schematic of adhesion assay. α4β7 -expressing RPMI8866 cells (yellow) were incubated in the absence or presence of anti-α4β7 or -β7 mAbs (Vedolizumab, 2B4) (dark blue) and then added to plates previously coated with the cV2 peptide (purple). Alternatively, cV2 peptides are pre-incubated with anti-V2 specific mAbs (green). Plates were washed and AlamarBlue dye was added to each well. Fluorescence was measured for 8 hours at 1-hour intervals (OD590nm). B) Expression and distribution of β7 on RPMI8866 cells +/- RA, stained with an anti-β7 PE mAb or an IgG2a-PE isotype control mAb viewed by confocal microscopy. Upper panels: differential interference contrast (DIC), lower panels: fluorescence (red). C) Adhesion of RPMI8866 cells, cultured in the presence (+RA) or absence (-RA) of retinoic acid, to MAdCAM-Ig, or cyclic V2 peptides derived from HIV 92TH023, C06980v0c22, and BG505. Adhesion was determined by OD590nm and listed as fluorescence units (y-axis). Background fluorescence (BF) of RPMI8866 cell adhesion to a blank well is denoted by a dashed line. (TIF) [file ppat.1007278.s002.tif]

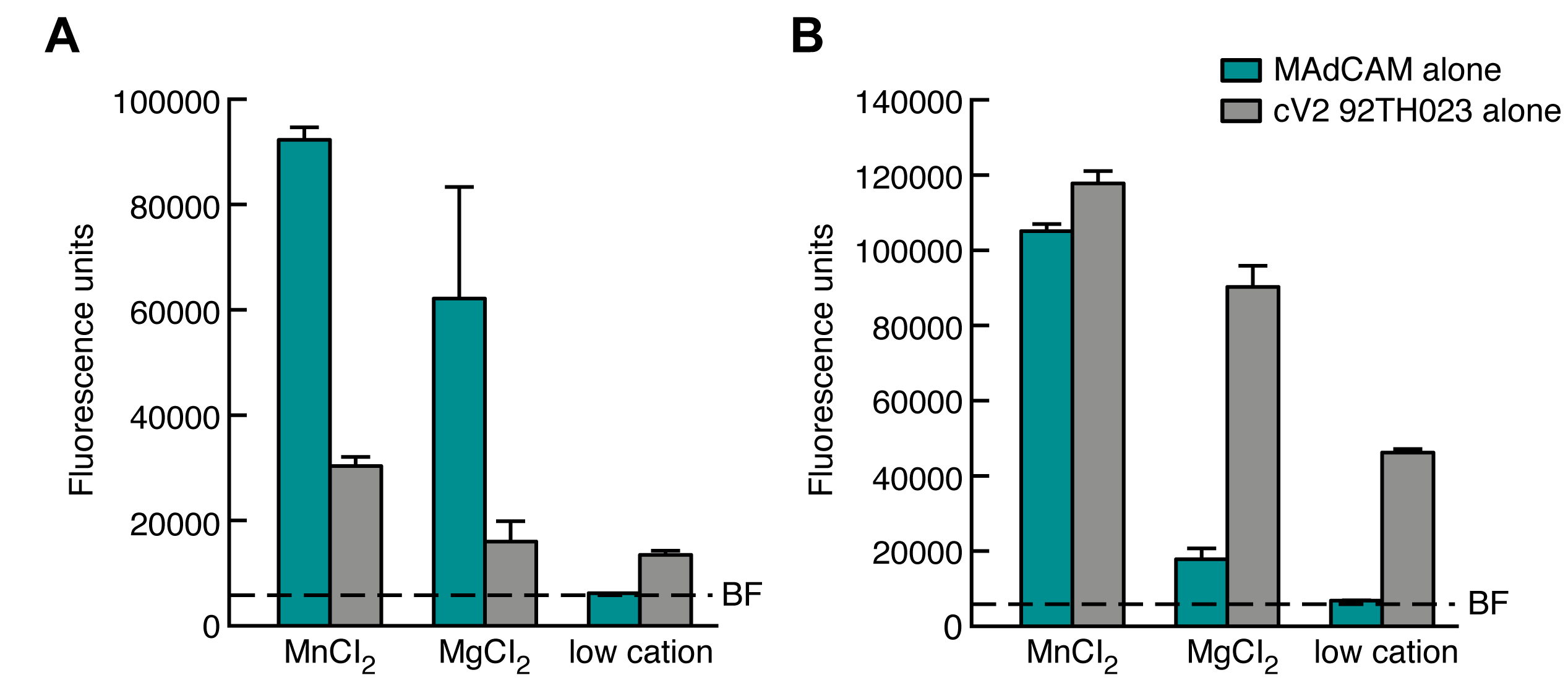

Supplement: S3 Fig — A-B) Adhesion of RPMI8866 cells to immobilized MAdCAM or a cV2 92TH023 peptide in the buffers containing a low concentration of divalent cations, or high concentrations of MnCl2 or MgCl2 as reported in Fig 2F in two additional independent experiments. Adhesion was determined at OD590nm and listed as fluorescence units (y-axis). Conditions are run in triplicate and error bars indicate standard error of the mean (SEM). Background fluorescence (BF) of RPMI8866 cells to blank wells is denoted by a dashed line. (TIF) [file ppat.1007278.s003.tif]

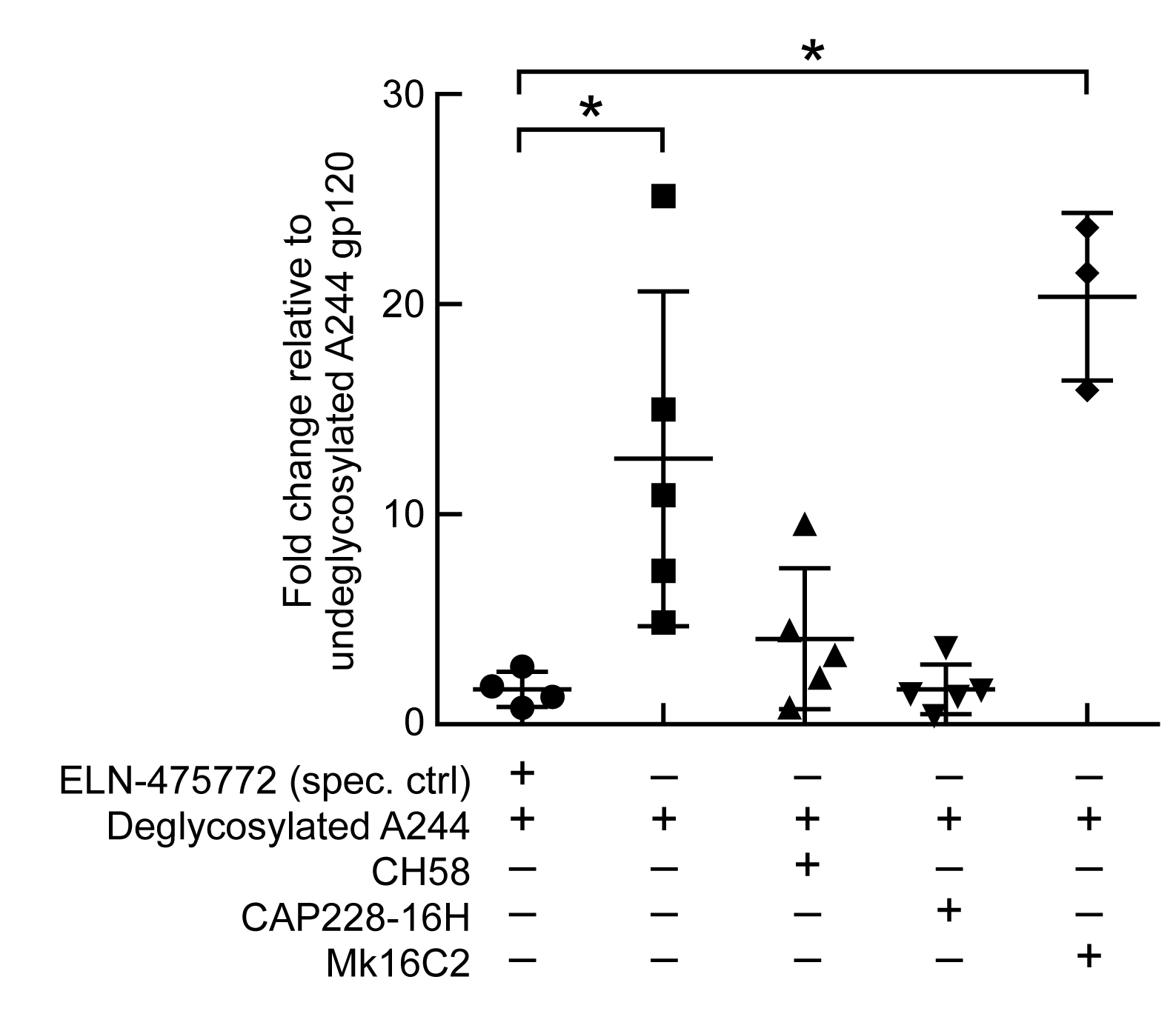

Supplement: S4 Fig — Adhesion of RPMI8866 cells to immobilized deglycosylated A244 gp120 in presence of HIV V2-specific mAbs: CH58, CAP228-16H, and Mk16C2. The LDV mimetic ELN-475772 was included as a specificity control (spec. ctrl) for adhesion to α4β7. Average adhesion in three or more independent experiments is reported as fold-change in adhesion relative to undeglycosylated A244 gp120 (y-axis). Error bars indicate SD. Significance determined by unpaired t-test (*p <0.05). (TIF) [file ppat.1007278.s004.tif]

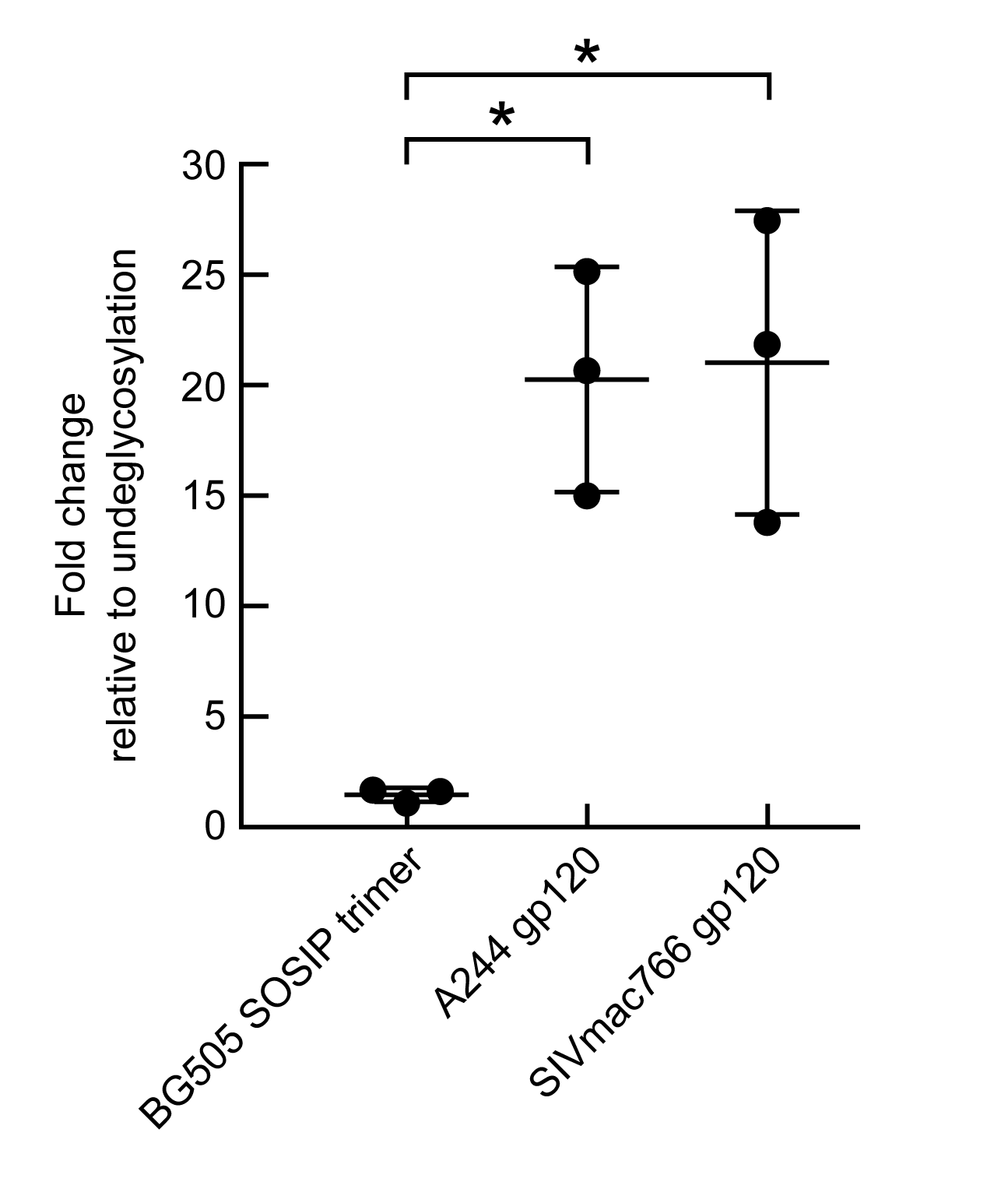

Supplement: S5 Fig — Adhesion of RPMI8866 cells to immobilized DG forms of BG505 SOSIP trimer, A244 gp120, and SIVmac766 gp120 relative to corresponding fully glycosylated forms of each protein expressed as fold-change (y-axis). Results from three independent experiments are shown. Error bars indicate SD. Significance determined by unpaired t-test (*p <0.05). (TIF) [file ppat.1007278.s005.tif]

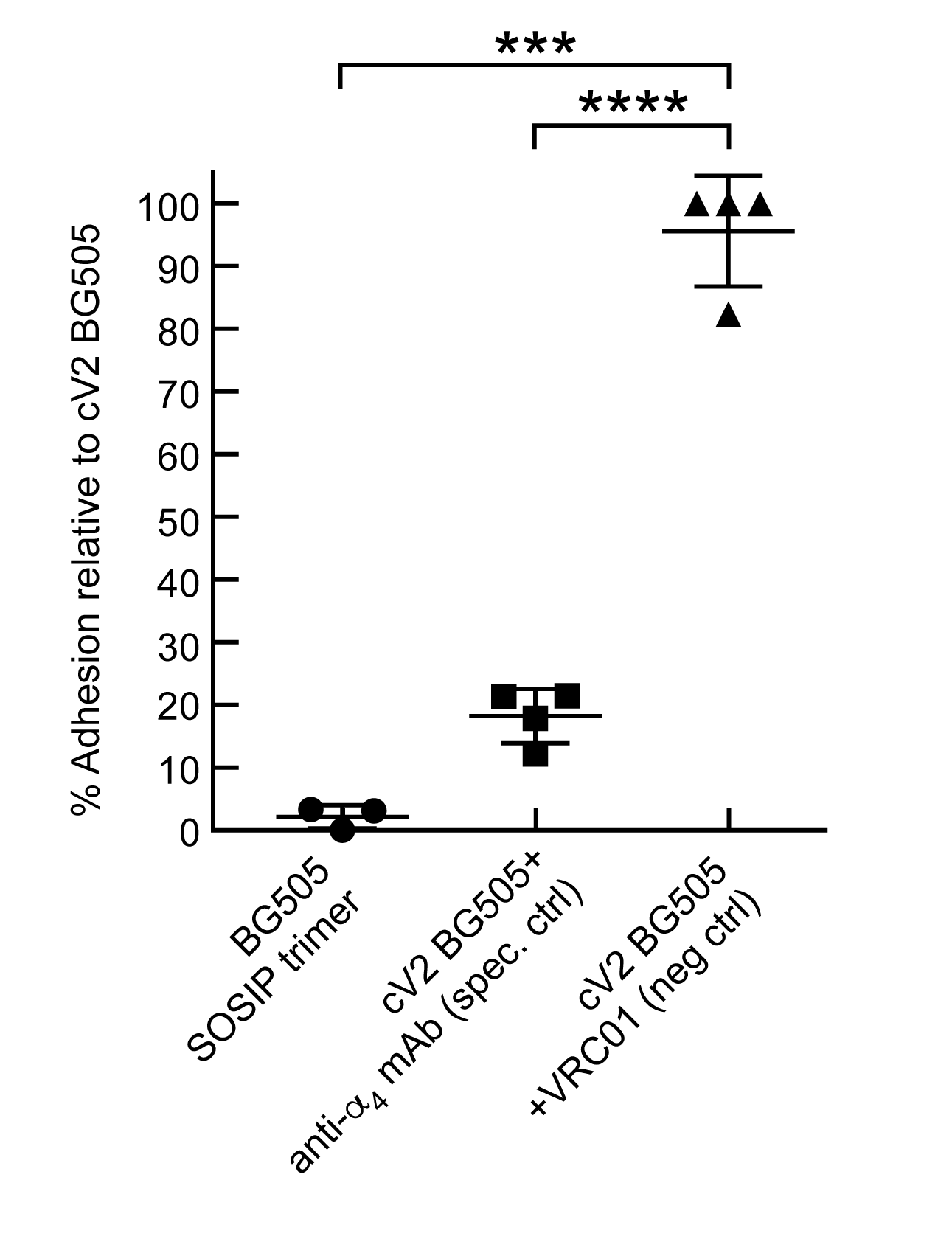

Supplement: S6 Fig — Adhesion of RPMI8866 cells to BG505 SOSIP trimer or cV2 BG505. Results from three or more independent experiments are shown and reported as % adhesion relative to cV2 BG505 in the absence of any inhibitor or in the presence of a specific inhibitor. The anti-α4 mAb 2B4 which was employed as a specificity control (spec. ctrl) for cV2 BG505, and VRC01 was employed as a nonspecific mAb control for cV2 BG505. Error bars indicate SD. Significance determined by unpaired t-test (***p <0.001 and ****p <0.0001). (TIF) [file ppat.1007278.s006.tif]

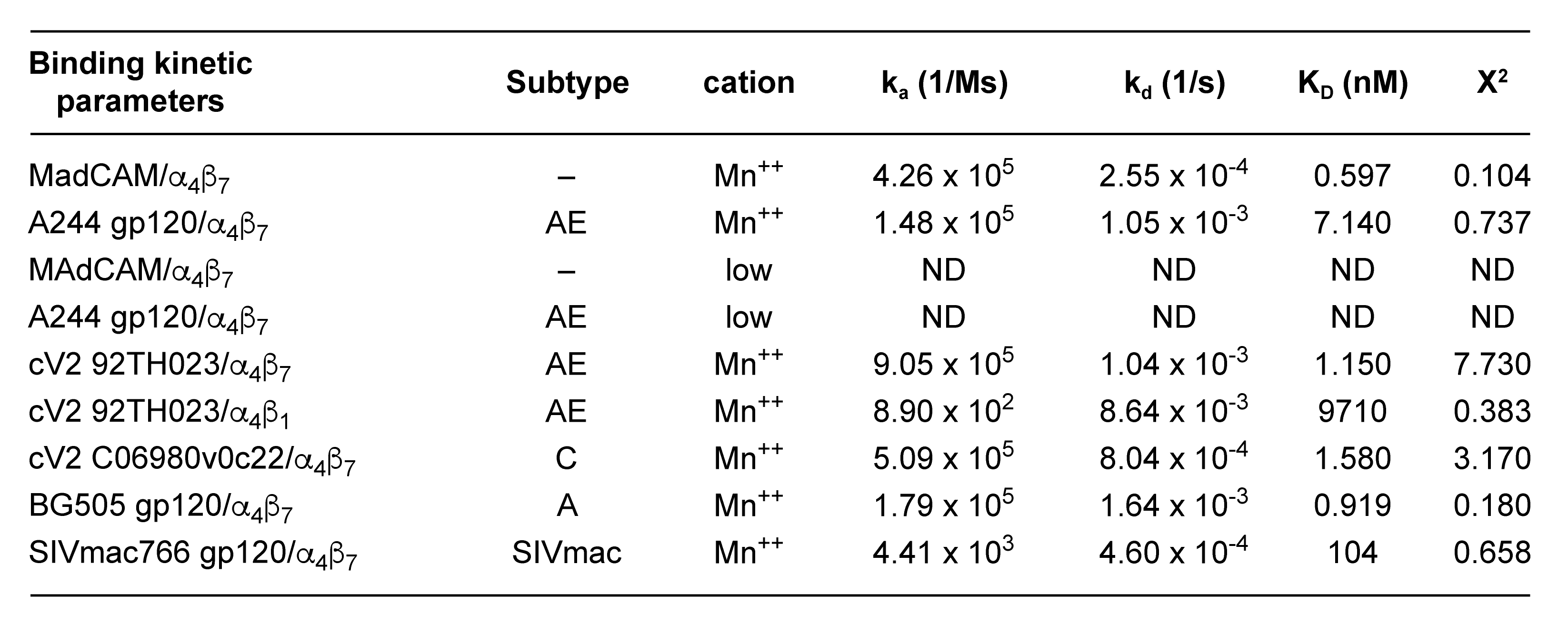

Supplement: S1 Table — (TIF) [file ppat.1007278.s007.tif]

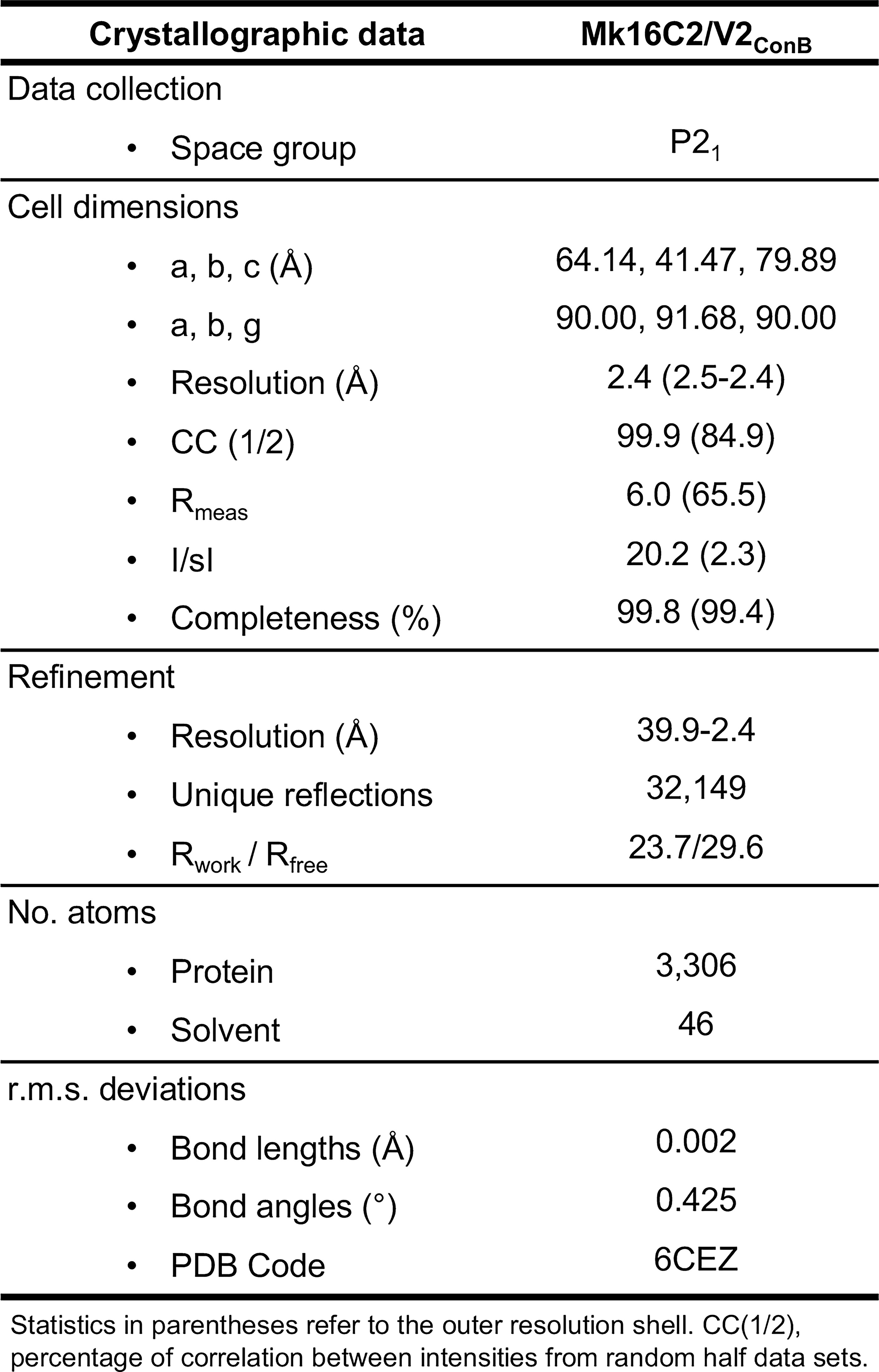

Supplement: S2 Table — (TIF) [file ppat.1007278.s008.tif]
